# Validation and test–retest repeatability performance of parametric methods for [^11^C]UCB-J PET

**DOI:** 10.1186/s13550-021-00874-8

**Published:** 2022-01-24

**Authors:** Hayel Tuncel, Ronald Boellaard, Emma M. Coomans, Marijke den Hollander-Meeuwsen, Erik F. J. de Vries, Andor W. J. M. Glaudemans, Paula Kopschina Feltes, David Vállez García, Sander C. J. Verfaillie, Emma E. Wolters, Steven P. Sweeney, J. Michael Ryan, Magnus Ivarsson, Berkley A. Lynch, Patrick Schober, Philip Scheltens, Robert C. Schuit, Albert D. Windhorst, Peter P. De Deyn, Bart N. M. van Berckel, Sandeep S. V. Golla

**Affiliations:** 1 Department of Radiology and Nuclear Medicine, Amsterdam Neuroscience, Amsterdam UMC, De Boelelaan 1117, 1081 HV Amsterdam, The Netherlands; 2 Department of Nuclear Medicine and Molecular Imaging, University Medical Center, University of Groningen, Groningen, The Netherlands; 3 Alzheimer Center Amsterdam, Department of Neurology, Amsterdam Neuroscience, Amsterdam UMC, Amsterdam, The Netherlands; 4 Rodin Therapeutics Inc., Cambridge, MA USA; 5 Department of Anaesthesiology, Amsterdam UMC, Amsterdam, The Netherlands; 6 Department of Neurology, University of Groningen, University Medical Center Groningen, Groningen, The Netherlands; 7 Alzheimer Research Center, University of Groningen, University Medical Center Groningen, Groningen, The Netherlands

**Keywords:** Alzheimer’s disease, [^11^C]UCB-J, Parametric methods, PET, SV2A

## Abstract

[^11^C]UCB-J is a PET radioligand that binds to the presynaptic vesicle glycoprotein 2A. Therefore, [^11^C]UCB-J PET may serve as an in vivo marker of synaptic integrity. The main objective of this study was to evaluate the quantitative accuracy and the 28-day test–retest repeatability (TRT) of various parametric quantitative methods for dynamic [^11^C]UCB-J studies in Alzheimer’s disease (AD) patients and healthy controls (HC). Eight HCs and seven AD patients underwent two 60-min dynamic [^11^C]UCB-J PET scans with arterial sampling over a 28-day interval. Several plasma-input based and reference-region based parametric methods were used to generate parametric images using metabolite corrected plasma activity as input function or white matter semi-ovale as reference region. Different parametric outcomes were compared regionally with corresponding non-linear regression (NLR) estimates. Furthermore, the 28-day TRT was assessed for all parametric methods. Spectral analysis (SA) and Logan graphical analysis showed high correlations with NLR estimates. Receptor parametric mapping (RPM) and simplified reference tissue model 2 (SRTM2) BP_ND_, and reference Logan (RLogan) distribution volume ratio (DVR) regional estimates correlated well with plasma-input derived DVR and SRTM BP_ND_. Among the multilinear reference tissue model (MRTM) methods, MRTM1 had the best correspondence with DVR and SRTM BP_ND_. Among the parametric methods evaluated, spectral analysis (SA) and SRTM2 were the best plasma-input and reference tissue methods, respectively, to obtain quantitatively accurate and repeatable parametric images for dynamic [^11^C]UCB-J PET.

**Supplementary Information:**

The online version contains supplementary material available at 10.1186/s13550-021-00874-8.

## Background

Abnormal brain deposits of amyloid β (Aβ), aggregation of tau into neurofibrillary tangles (NFTs) and synaptic loss are neuropathological hallmarks of Alzheimer’s disease (AD) [1]. According to new AD models, the accumulation of abnormal proteins and synaptic loss occur many years before the onset of AD [2, 3]. Previous studies reported that lower synaptic density in the hippocampus and cerebral cortex is associated with cognitive impairment in AD patients [4, 5]. In these papers synaptic density was measured post-mortem; however, recently, it became possible to quantify synaptic density *in-vivo* using PET with the ligand [^11^C]UCB-J which targets the synaptic vesicle protein 2A (SV2A).

SV2A is a glycoprotein present in the membrane of presynaptic vesicles and is located in synapses throughout the brain. There are three different isoforms of synaptic vesicle proteins: SV2A, SV2B and SV2C [6]. SV2 proteins are involved in vesicle transport in the synapse and are essential for the function of our nervous system. Although the specific physiological role of SV2A is still unclear, SV2A is thought to be involved in the exocytosis of neurotransmitters and plays an important role in regulating/modulating synaptic function^6^. Since SV2A is highly expressed throughout the brain, it is a suitable target for positron emission tomography (PET) imaging when aiming to assess synaptic integrity in vivo.

(R)-1-((3-([^11^C]methyl)pyridin-4-yl)methyl)-4-(3,4,5-trifluorophenyl)pyrrolidin-2-one ([^11^C]UCB-J) is a PET ligand with high affinity and specificity for SV2A [7, 8]. [^11^C]UCB-J enables in vivo imaging of synaptic density in neurological diseases. Previous studies have found that [^11^C]UCB-J kinetics can be best described by a reversible one-tissue compartment model with additional blood volume fraction as a parameter (1T2k_V_B_) [9, 10]. Moreover, the white matter semi-ovale (SO), has been validated as a suitable reference region for quantification of synaptic density in the brain [11].

To date, the (regional) kinetics of [^11^C]UCB-J have mainly been assessed using conventional tracer kinetic models based on predefined regions of interest (ROI). In addition to ROI approaches, voxel-wise analysis, using quantitative parametric images can give additional information when a specific signal is not homogenous in a region and gets diluted when evaluated at a regional level. Therefore, it is important to obtain quantitatively accurate parametric maps. In a recent study evaluating various parametric methods for dynamic [^11^C]UCB-J PET, Mertens et al. reported that the simplified reference tissue model 2 (SRTM2) was the preferred parametric method for voxel-wise analysis using white matter SO as reference region [12]. However, this study was performed only in healthy controls (HCs) and no test–retest repeatability (TRT) data was assessed to evaluate the performance of the parametric methods. In a recent other study [10], we assessed TRT at regional level for conventional kinetic models for [^11^C]UCB-J in a 28-day time period in order to closely mimic the condition of the clinical drug intervention design [13]. The expected effect size was 25% in a 28-day time period [13]. The aim of the current study was to validate various parametric methods to obtain quantitatively accurate [^11^C]UCB-J parametric images in both HCs and mild to moderate AD patients. In addition, to assess the TRT for these parametric methods over a 28-day period in order to closely mimic the clinical drug intervention design.

## Methods

### Participants

Eight HCs with an average age of 63.1 ± 6.3 years and MMSE score of 29.4 ± 0.9 and seven mild to moderate AD patients with an average age of 64.3 ± 8.3 years and MMSE score of 24.1 ± 1.8 from the Amsterdam University Medical Center (Amsterdam UMC) participated in the study. All AD patients had a diagnosis of probable AD defined by National Institute on Aging—Alzheimer’s Association (NIA-AA) [14]. All AD patients had positive Aβ biomarkers either determined by Aβ_42_ in cerebrospinal fluid (CSF) (Aβ_42_ < 813 pg/mL) [15]) or visual read of an amyloid-β PET scan, and a Mini-Mental State Examination (MMSE) score between 18–26. All HCs were cognitively normal without cognitive complaints, absence of significant impairment in cognitive functions or activities of daily living and the MMSE score was ≥ 27. The Medical Review and Ethics Committee (MREC) of Foundation BEBO in Assen approved the current study and local feasibility was confirmed by the MREC of Amsterdam UMC. Furthermore, all subjects had to provide written informed consent prior to enrolment.

### Data acquisition

3D T1 weighted MRI scans were obtained using a 3.0 T Philips Ingenuity Time-of-Flight PET/MR scanner at Amsterdam UMC for all participants for brain tissue segmentation and PET co-registration. Each participant underwent two dynamic PET scans on the same PET/CT system with an interval of 28 days. The mean interval and SD between the test and retest scans was 28.3 ± 1.3 days. PET scans were acquired on the Ingenuity TF PET/CT scanner (Amsterdam UMC, Philips Medical Systems, Best, the Netherlands). In short, prior to each PET scan, a low-dose computed tomography (CT) was acquired for attenuation correction. The low-dose CT was followed by a dynamic PET scan after a bolus injection of 347 MBq ± 41 [^11^C]UCB-J. During scanning, the head of the subjects was stabilized to reduce movement artefacts. More specifically, subjects were positioned within the center of axial and transaxial fields of view, such that the orbito-meatal line was parallel to the detectors with the use of laser beams, this way motion during the PET acquisition was checked every now and then and was minimized as much as possible. The scan duration was 90 min for HC and 60 min for AD patients. Based on the results of previous studies [9, 10], only 60 min scan data will be used for the present study. The PET list mode data were rebinned into a total of 19 frames (1 × 15, 3 × 5, 3 × 10, 4 × 60, 2 × 150, 2 × 300 and 4 × 600 s) followed by a reconstruction using 3D RAMLA with a matrix size of 128 × 128 × 90 and a final voxel size of 2 × 2 × 2 mm^3^, including all usual corrections for dead time, decay, attenuation, randoms and scatter.

Continuous arterial blood sampling using an online detection system [16] was acquired continuously over 30 min for AD patients and HCs. At set time points (5, 10, 15, 20, 40, 50, 60 for AD patient and, 5, 10, 15, 20, 40, 50, 60, 75 and 90 min for HCs) manual blood samples were collected (5–7 mL each), to estimate the plasma-to-whole-blood ratios and to measure plasma metabolite fractions. The continuous online blood sampler data were calibrated using the manual whole blood activity and corrected for metabolites, plasma to whole blood ratios and delay, using information measured from manual samples. This resulted in a metabolite corrected arterial plasma input function. Manual blood samples were collected in heparin tubes and centrifuged for 5 min at 5000 r/min.

### Data analysis

#### Image processing

The 3D T1 weighted MR images were co-registered onto the dynamic PET scan using VINCI v 2.56 software (Max Plank Institute, Cologne, Germany). Sixty eight regions of interest (ROIs) as in Hammers template [17] were defined on the co-registered MRI using PVElab [18]. Corresponding regional time activity curves (TACs) were extracted by superimposing these 68 ROIs onto the dynamic PET scan.

#### Kinetic analysis (non-linear regression)

Earlier studies have determined that a 1T2k_V_B_ best describes the in vivo kinetics of [^11^C]UCB-J [9, 10]. Therefore, 1T2k_V_B_ model was used to estimate the kinetic parameters such as, distribution volume ratio (DVR), total volume of distribution (V_T_) and rate of influx of radioligand from blood to tissue (K_1_). The regional non-linear regression (NLR) estimates were obtained with a home-developed MATLAB script and served as a gold standard to evaluate settings and validate different parametric methods. Furthermore, reference tissue-based parametric methods were validated against corresponding kinetic parameters, such as binding potential (BP_ND_) and R_1_ (influx of the tracer into the ROI relative to the reference region) derived by using simplified reference tissue model (SRTM). White matter SO was considered as the reference region.

#### Parametric images

Parametric images were obtained by using an in-house built software tool (PPET) [19]. Various linearization methods were evaluated: plasma input Logan (Logan) [20], reference Logan (RLogan) [21] and five versions of the multilinear reference tissue model (MRTMo, MRTM1, MRTM2, MRTM3, MRTM4) [22–24]. Furthermore, basis function methods such as receptor parametric mapping (RPM) [25], SRTM2 [26] and spectral analysis (SA) [27] were validated. Plasma input based Logan and SA were studied to generate V_T_ and/or K_1_ images. To produce the BP_ND_ and R_1_ images, RLogan, MRTMo-4, RPM and SRTM2 were evaluated. For SA, a linear regression fitting model (weighted residual sum of squares) was used. Equation 1 illustrates the weighting factors estimation [28].

1 $$\sigma^{2} = dcf \cdot dcf \frac{{{T}}}{{{{L }} \cdot { }L}} .$$

The outcome $\sigma^{2}$ represents the variances calculated for each frame and is based on the whole scanner true counts (T), decay correction factor ($dcf$) and frame length ($L$). Whole scanner true counts were obtained from the scanner acquisition statistics, or estimated using (not-corrected) total counts in each frame. The decay correction factor was calculated using the formula stated by Yaqub et al. [28] and frame length (in seconds) is an acquisition parameter. Weighting factors for each frame were calculated using $1/\sigma^{2}$. The above mentioned models were fitted to the data using non-linear least squares in combination with these weighting factors.

Different start times (t*) were evaluated by comparing the estimated regional parametric values to the reference standard (parametric values estimated using plasma input based 1T2k_V_B_ model) for linearization methods. However, in case of parametric methods using basis function approach, an initial estimate of the θ_1_, θ_2_, and θ_3_, was derived using the equations described by Gunn et al. 1997 [25]. These values were further adapted to obtain the optimal settings by comparing the regional parametric estimates to the reference standard. Furthermore, the weighting factors used in the implementation of the parametric methods were based on the study by Yaqub et al. [28].

### Statistical analysis

To evaluate the correspondence between parametric methods estimates and NLR estimates across all ROIs and across all subjects, coefficients of determination (*r*^2^) and the slopes of the regression line were calculated. Equation 2 was used to calculate the TRT for each parametric method in native space for regional values. Furthermore, all parametric images were warped to Montreal Neurological Institute (MNI152) space, with Statistical Parametric Mapping (SPM) version 12 software (Welcome Trust Center for Neuroimaging, University College London, UK). Transformation matrixes derived from warping the co-registered MRI scans to MNI were used for this purpose. The warped images were used to calculate absolute TRT repeatability (average and SD) using Eq. 3 for each parametric method. The bias to assess overestimation or underestimation was calculated using Eq. 4. Furthermore, the intraclass correlation coefficient (ICC) was analysed using an average-measurement, absolute-agreement, 2-way mixed-effects model for each parametric method.

2 $${\text{TRT}}\;(\% ) \, = \frac{{\left( {{\text{Retest}}\;{\text{value}} - {\text{Test}}\;{\text{value}}} \right) }}{{\left( {{\text{Retest }}\;{\text{value}} + {\text{Test}}\;{\text{value}}} \right) }} \times 200.$$

3 $${\text{Absolute}}\;{\text{TRT}}\;{(}\% {) } = \frac{{\left| {\left( {{\text{Retest }}\;{\text{value}} - {\text{Test }}\;{\text{value}}} \right)} \right|{ }}}{{\left( {{\text{Retest }}\;{\text{value}} + {\text{Test }}\;{\text{value}}} \right){ }}} \times 200.$$

4 $${\text{Bias}}\;{(}\% {) } = \frac{{\left( {{\text{Parametric }}\;{\text{value}} - {\text{Conventional }}\;{\text{method }}\;{\text{value}}} \right){ }}}{{{\text{Conventional}}\;{\text{ method}}\;{\text{ value}}}} \times 100.$$

## Results

**Fig. 1:**
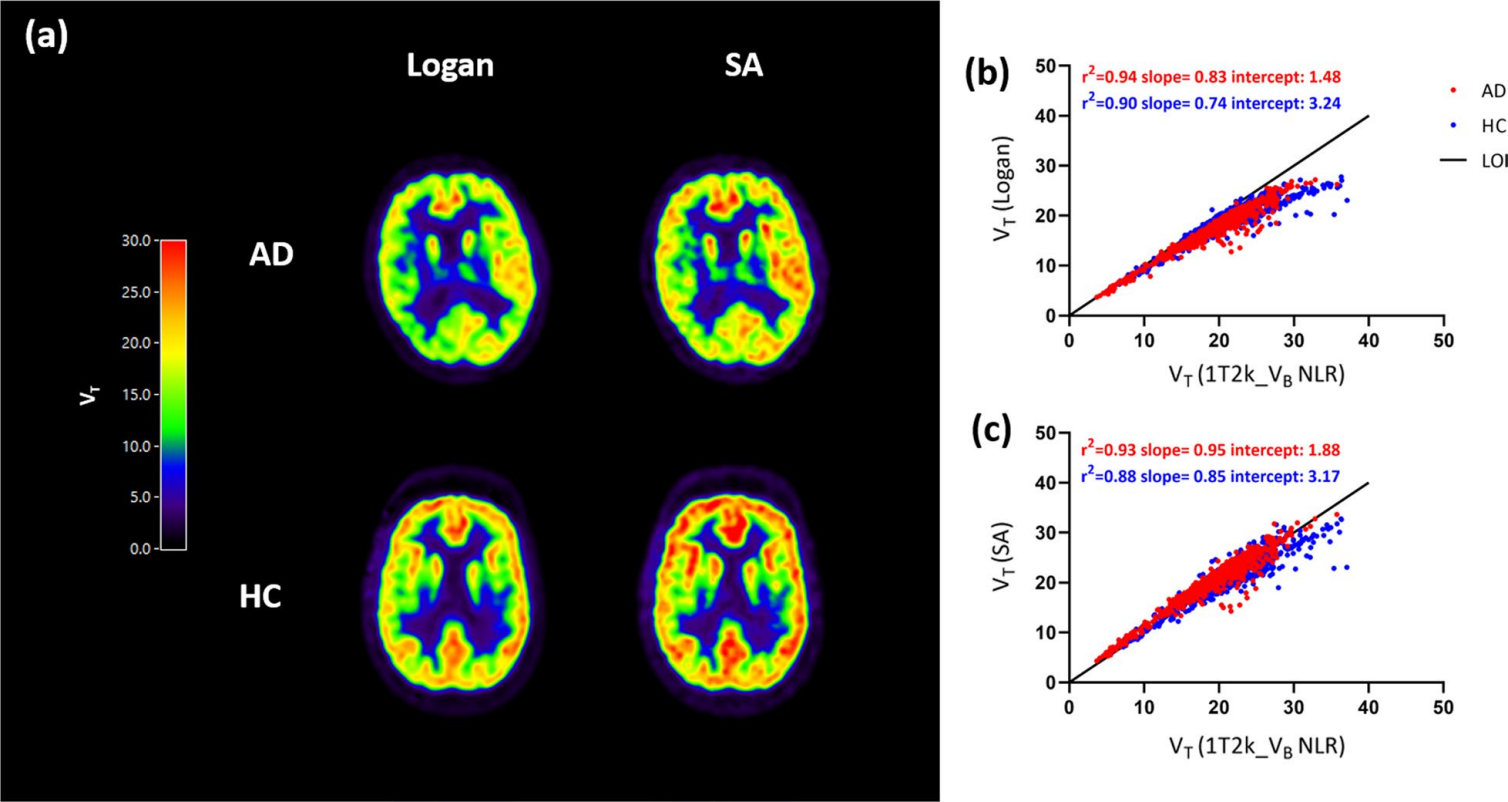
**a** Logan and SA derived V_T_ parametric images for a typical AD patient and HC subject. Coefficients of determination for regional V_T_ estimates of all Hammers template regions for all the subjects obtained using **b** Logan and **c** SA with corresponding NLR V_T_ estimates. AD: Alzheimer’s disease; HC: healthy control; LOI: line of identity

The optimal settings for the various parametric imaging methods are presented in Table 1. Parametric images for V_T_ derived from Logan and SA are presented in Fig. 1a, separately for a typical AD and HC subject. Regional V_T_ values obtained from SA (AD: *r*^*2*^ = 0.93, slope = 0.95; HC: *r*^*2*^ = 0.88, slope = 0.85) and Logan (AD: *r*^*2*^ = 0.94, slope = 0.83; HC: *r*^*2*^ = 0.90, slope = 0.74) had a good correspondence with V_T_ values derived from 1T2k_V_B_ using test and retest data (Fig. 1b, c). Coefficients of determination and slopes for SA and Logan V_T_ with corresponding NLR estimates are presented in Additional file 1: Fig. 1, separately for test and retest data for each group. Furthermore, scatterplots for V_T_ obtained with SA with corresponding NLR V_T_ estimates are presented in Additional file 2: Fig. 2, separately color-coded for each subject. In Additional files 3, 4: Figs. 3 & 4, scatterplots for V_T_ and K_1_ obtained with SA with corresponding NLR V_T_ and K_1_ estimates are presented, separately color-coded for each region. K_1_ estimates obtained from SA showed an underestimation of 14.1% ± 4.7 for AD patients and 13.2% ± 4.9 for HCs when compared to NLR derived K_1_ estimates (Additional file 12: Table 1). Coefficients of determination and slopes for each subject for parameters obtained from SA are presented in Additional file 13: Table 2.

**Table 1:** The optimal settings used for different parametric methods

Parametric method	Interval (min)	Basis function range (min^−1^)	Number of basis functions
Spectral analysis^a^	0–60	0.01–0.1	50
Logan^a^	10–60	–	–
Reference Logan^b^	30–60	–	–
RPM^b^	0–60	0.01–0.1	30
SRTM2^b^	0–60	0.01–0.1	30
MRTMo^b^	10–60	–	–
MRTM1^b^	10–60	–	–
MRTM2^b^	10–60	–	–
MRTM3^b^	10–60	–	–
MRTM4^b^	10–60	–	–

MRTM: multilinear reference tissue model; RPM: receptor parametric mapping; SRTM2: simplified reference tissue model 2. White matter semi-ovale was used as reference region

^a^Plasma input based implementations

^b^Reference tissue based implementations

**Fig. 2:**
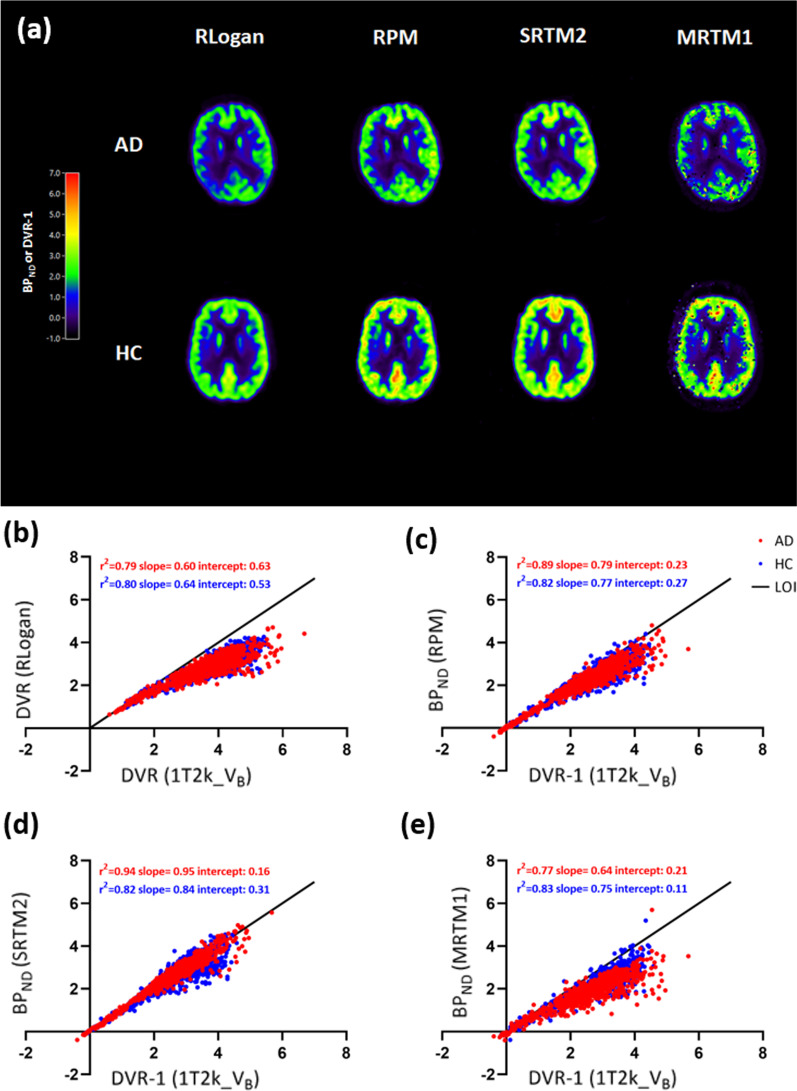
**a** RLogan, RPM, SRTM2, and MRTM1 DVR/BP_ND_ parametric images for a typical AD patient and HC. Scatterplots for regional DVR/BP_ND_ estimates of all Hammers template regions for all the subjects obtained using **b** RLogan, **c** RPM, **d** SRTM2, and **e** MRTM1 with corresponding NLR estimates. AD: Alzheimer’s disease; HC: healthy control; LOI: line of identity; BP_ND_: binding potential; DVR: distribution volume ratio; NLR: non-linear regression; RLogan: reference Logan; RPM: receptor parametric mapping; SRTM2: simplified reference tissue model 2

Figure 2a shows the DVR and BP_ND_ parametric images derived from RLogan, RPM, SRTM2 and MRTM1 implementations. RLogan, RPM and STRM2 provided visually good quality parametric images.

Coefficients of determination (*r*^*2*^) and slopes for each parametric implementation with their corresponding NLR estimates using 60 min data are presented in Table 2, 3, separately for AD patients and HCs. RLogan derived DVR correlated well with NLR derived DVR (AD: *r*^*2*^ = 0.79, slope = 0.60; HC: *r*^*2*^ = 0.80, slope = 0.64) (Fig. 2b) and SRTM BP_ND_ (AD: *r*^*2*^ = 0.83, slope = 0.75; HC: *r*^*2*^ = 0.82, slope = 0.78). RPM BP_ND_ correlated well with indirect measure of BP_ND_ (DVR-1) (AD: *r*^*2*^ = 0.89, slope = 0.79; HC: *r*^*2*^ = 0.82, slope = 0.77) (Fig. 2c) and SRTM BP_ND_ (AD: *r*^*2*^ = 0.97, slope = 1.02; HC: *r*^*2*^ = 0.92, slope = 1.00) using test and retest data. SRTM2 BP_ND_ showed the best correlations with DVR-1 (AD: *r*^*2*^ = 0.94, slope = 0.95; HC: *r*^*2*^ = 0.82, slope = 0.84) (Fig. 2d) and SRTM BP_ND_ (AD: *r*^*2*^ = 0.91, slope = 1.18; HC: *r*^*2*^ = 0.81, slope = 1.02). Furthermore, R_1_ obtained from both RPM (AD: *r*^*2*^ = 0.99, slope = 0.99; HC: *r*^*2*^ = 0.99, slope = 1.01) and SRTM2 (AD: *r*^*2*^ = 0.97, slope = 1.01; HC: *r*^*2*^ = 0.98, slope = 1.01) had an excellent correlation with NLR derived SRTM R_1_. Coefficients of determination (*r*^*2*^) and slopes for parameters obtained from RPM and SRTM2 are presented in Additional file 13: Table 2 and Additional file 14: Table 3 for all subjects separately to illustrate inter-subject variability. Furthermore, the % bias (mean + SD) of micro/macro-parameters estimated using the parametric methods of interest with respect to the corresponding 1T2k_V_B_ and SRTM estimates are presented in Additional file 12: Table 1 and Additional file 15: Table 4.

**Table 2:** Coefficients of determination (r^2^) and slope of parametric [^11^C]UCB-J V_T_, K_1_ and BP_ND_ against corresponding 1T2k_V_B_ estimates using 60 min data

	HC		AD	
	*r*^2^	Slope	*r*^2^	Slope
Spectral analysis^a^ V_T_	0.88	0.85	0.93	0.95
Spectral analysis ^a^ K_1_	0.94	0.85	0.97	0.84
Logan ^a^ V_T_	0.90	0.74	0.94	0.83
Reference Logan^b^ DVR	0.80	0.64	0.79	0.60
RPM^b^ BP_ND_	0.82	0.77	0.89	0.79
SRTM2^b^ BP_ND_	0.82	0.84	0.94	0.95
MRTMo^b^ BP_ND_	0.79	0.62	0.76	0.57
MRTM1^b^ BP_ND_	0.83	0.75	0.77	0.64
MRTM2^b^ BP_ND_	0.59	0.78	0.40	1.13
MRTM3^b^ BP_ND_	0.75	0.64	0.63	0.51
MRTM4^b^ BP_ND_	0.85	0.81	0.56	0.87

BP_ND_: binding potential; MRTM: multilinear reference tissue model; NLR: non-linear regression; RPM: receptor parametric mapping; SRTM2: simplified reference tissue model 2.

^a^Plasma input implementations

^b^Reference region implementations

**Table 3:** Coefficients of determination (r^2^) and slope of parametric [^11^C]UCB-J DVR, BP_ND_ and R_1_ against corresponding SRTM estimates

	HC		AD	
	*r*^2^	Slope	*r*^2^	Slope
Reference Logan^b^ DVR	0.82	0.78	0.83	0.75
RPM^b^ BP_ND_	0.92	1.00	0.97	1.02
RPM^b^ R_1_	0.99	1.01	0.99	0.99
SRTM2^b^ BP_ND_	0.81	1.02	0.91	1.18
SRTM2^b^ R_1_	0.98	1.01	0.97	1.01
MRTMo BP_ND_^b^	0.81	0.76	0.85	0.73
MRTMo ^b^ R_1_	0.40	0.70	0.34	0.67
MRTM1^b^ BP_ND_	0.85	0.91	0.87	0.83
MRTM1^b^ R_1_	0.93	1.09	0.91	1.02
MRTM2^b^ BP_ND_	0.53	0.89	0.43	1.41
MRTM2^b^ R_1_	0.93	1.10	0.94	1.02
MRT3^b^ BP_ND_	0.80	0.80	0.70	0.66
MRTM3^b^ R_1_	0.02	0.11	0.00	0.05
MRTM4^b^ BP_ND_	0.87	1.01	0.60	1.06
MRTM4^b^ R_1_	0.05	0.08	0.00	− 0.06

BP_ND_: binding potential; MRTM: multilinear reference tissue model; NLR: non-linear regression; RPM: receptor parametric mapping; SRTM2: simplified reference tissue model 2

^a^Plasma input implementations

^b^Reference region implementations

Among the MRTM methods, MRTM1 had the best correspondence with DVR (AD: *r*^*2*^ = 0.77, slope = 0.64; HC: *r*^*2*^ = 0.83, slope = 0.75) (Fig. 2e) and SRTM BP_ND_ (AD: *r*^*2*^ = 0.87, slope = 0.83; HC: *r*^*2*^ = 0.85, slope = 0.91). Most of the MRTM parametric images were noisy and had qualitatively unreliable parametric images. Coefficients of determination and slopes for DVR and BP_ND_ obtained from RLogan, RPM, SRTM2 and MRTM1 with corresponding NLR estimates are presented in Additional file 5: Fig. 5, separately for test and retest data for each subject group. Furthermore, scatterplots for BP_ND_ estimates obtained using RPM and SRTM2 against corresponding NLR V_T_ estimates are presented in Additional files 6, 7: Figs. 6 & 7, separately color-coded for each subject. In addition, scatterplots of the parameters obtained from SRTM2 with corresponding NLR estimates are presented in Additional files 8, 9: Figs. 8 & 9, separately color-coded for each region.

The TRT of different parametric methods using 60 min data for whole brain grey matter are presented in Table 4 separately for AD patients and HCs. Most of the evaluated parametric methods had comparable TRTs as the NLR counterpart methods. TRT for a few other brain regions for parameters estimates using SA, SRTM2, and RPM are presented in Additional files 15, 16, 17, 18: Tables 4, 5, 6, 7. ICC for each parametric method and associated parametric estimation(s) are presented in Table 5. In addition, the TRT voxel-wise images (average and SD) calculated for HCs and AD patients are presented in Additional files 10, 11: Figs. 10 & 11.

**Table 4:** TRT values estimated for whole brain (grey matter) are presented for each parametric method

TRT whole brain (grey matter)				
	HC		AD	
	Mean	SD	Mean	SD
SA V_T_	− 9	7	2	9
SA K_1_	1	12	6	13
Logan V_T_	− 5	5	3	8
RLogan DVR	− 3	6	6	9
RPM DVR (BP_ND_ + 1)	− 5	8	6	13
RPM R_1_	− 3	7	3	11
SRTM2 DVR (BP_ND_ + 1)	− 2	10	6	10
SRTM2 R_1_	− 2	7	3	9
MRTM1 DVR (BP_ND_ + 1)	− 4	6	8	13
MRTM1 R_1_	0	12	1	10

TRT for NLR V_T_ and K_1_ was − 8% ± 4., − 2% ± 14 for HCs and 3% ± 8, 3% ± 14 for AD patients, respectively. TRT for Plasma-input DVR was − 7% ± 6 for HCs and 7% ± 13 for AD patients. TRT for SRTM derived DVR (BP_ND_ + 1) and R_1_ was − 6% ± 7, − 2% ± 6 for HCs and was − 5% ± 9, 2% ± 10 for AD patients, respectively

**Table 5:** ICC values estimated for each parametric method and associated parametric estimation(s) separately for HC’s and AD patients

	HC		AD	
	ICC	95% CI	ICC	95% CI
SA V_T_	0.97	0.95–0.98	0.98	0.96–0.99
SA K_1_	0.97	0.91–0.98	0.95	0.92–0.97
Logan V_T_	0.98	0.97–0.99	0.98	0.96–0.98
RLogan DVR	0.96	0.92–0.98	0.96	0.94–0.98
RPM BP_ND_	0.96	0.93–0.98	0.96	0.94–0.98
RPM R_1_	0.96	0.93–0.98	0.96	0.93–0.97
SRTM2 BP_ND_	0.97	0.95–0.98	0.97	0.95–0.98
SRTM2 R_1_	0.97	0.94–0.98	0.96	0.93–0.97
MRTM1 BP_ND_	0.96	0.93–0.97	0.95	0.92–0.97
MRTM1 R_1_	0.95	0.91–0.97	0.96	0.93–0.97

SA, RPM and SRTM2 parametric implementations were also evaluated using 90 min data for HCs only. Coefficients of determination (*r*^*2*^) and slopes for these parametric implementations with their corresponding NLR estimates using 90 min data are presented in Additional files 19, 20: Tables 8 & 9. Regional V_T_ values obtained from SA (HC: *r*^*2*^ = 0.90, slope = 0.84) had a good correspondence with V_T_ values derived from 1T2k_V_B_. Furthermore, RPM BP_ND_ correlated well with DVR-1 (HC: *r*^*2*^ = 0.90, slope = 0.79) and SRTM BP_ND_ (HC: *r*^*2*^ = 0.97, slope = 0.99). SRTM2 BP_ND_ showed the good correlations with DVR-1 (HC: *r*^*2*^ = 0.89, slope = 0.82) and SRTM BP_ND_ (HC: *r*^*2*^ = 0.93, slope = 1.00). The TRT of SA, RPM and SRTM2 parametric methods using 90 min data for whole brain grey matter are presented in Additional file 21: Table 10 for HCs.

## Discussion

Since disease-specific signal is not homogenous throughout the ROI, relying solely on regional analyses may potentially result in loss of significant signal differences due to spatial dilution. Therefore, voxel-level analyses are necessary to obtain quantitatively accurate parametric images. The present study showed that SA performed better than Logan and in case of reference region based implementations, SRTM2 performs the best for [^11^C]UCB-J quantification_._

V_T_ values obtained with SA proved to have a better correspondence with V_T_ values derived from 1T2k_V_B_ compared to Logan. Logan showed an underestimation when compared to regional NLR estimates. The underestimation either could be explained by statistical noise [27], or by the fact that Logan implementation does not account for blood volume fraction (V_B_). This bias was partly compensated by assuming that V_B_ is constant but since V_B_ varied between subjects and different brain regions, no V_B_ corrections was applied while performing the Logan analysis. Alternatively, SA is a plasma-input based basis function approach that also accounts for V_B_, the correspondence with NLR estimates is much better when compared to Logan. Moreover, SA also generates parametric images for K_1,_ which also had good correspondence with NLR K_1_ estimates (Table 2).

Regarding reference tissue methods, MRTMo-4, RLogan, RPM and SRTM2 were evaluated. For RLogan DVR, an underestimation of 21.1% ± 9.3 for AD patients and 20.7% ± 8.4 for HCs was observed when compared to plasma input (NLR) DVR and 9.7% ± 8.2 for AD patients and 8.8% ± 7.0 for HCs when compared to SRTM (NLR) BP_ND_. This could be explained by the noise induced negative biases of RLogan [29]. Normally, the disadvantage of using graphical methods such as RLogan is that the noise in the TACs is elevated due to a correlation of errors in the dependent and independent variable of the RLogan equation, which results in an underestimation of DVR. RPM BP_ND_ also presented an underestimation of 11.0% ± 10.8 for AD patients and 12.2% ± 12.9 for HCs when compared to plasma input (NLR) derived DVR, but had excellent correspondence with SRTM (NLR). In our recent study [10], a good correlation between regional estimates of SRTM (NLR) BP_ND_ and plasma input (NLR) DVR was observed but also with an underestimation of approximately 25% [10]. White matter SO showed different kinetics than the rest of the brain which could possibly explain this underestimation. Normally, an ideal reference region has a K_1_’/k_2_’ equal to the K_1_/k_2_ of the other brain regions, thus assuming that non-displaceable binding is equal between the reference region and other brain regions. Furthermore, an ideal reference region also has a faster efflux rate (k_2_’) than the apparent efflux rate of other brain regions (k_2a_ = k_2_/(1 + BP_ND_)), which makes the three parameters of interest (R_1_, k_2_’ and k_2a_) identifiable in the SRTM equation (see Eq. 5).

5 $${{ C}}\left( {{t}} \right){ } = {{ R}}_{1} {{C^{\prime}}}\left( {{t}} \right){ } + {{ R}}_{{1}} { }\left( {{{k}}_{{2}} {^{\prime} } - {{ k}}_{{{{2a}}}} } \right){{ C^{\prime} * exp}}\left( {{ } - {{k}}_{{{{2a}}}} {{ t}}} \right){ }{{.}}$$

For white matter SO, however, k_2_’ was equal to k_2a,_ which would make the (k_2_′–k_2a_) non identifiable. This might be the reason for SRTM not performing well for [^11^C]UCB-J, which could also imply that using plasma input (NLR) DVR an indirect measure of BP_ND_ is a better reference for parametric methods validation.

A recent study by Rossano et al. [30] observed differences in white matter kinetics and found approximately 20% higher non-displaceable uptake in the white matter compared to grey matter regions. This finding indicates that even though white matter is deprived of specific binding, [^11^C]UCB-J kinetics in the non-displaceable compartment may be different in white matter than in grey matter regions, which also makes the use of white matter SO as a reference region challenging. The differences in kinetics of white and grey matter could be explained by the differences in the tissue itself. Namely, grey matter is composed of neurons and glial cells, while white matter mainly consists of myelinated axons and oligodendroglia. White matter contains a higher concentration of lipids compared to grey matter due to the myelin that is present around the axons [31]. This could explain the lower k_2_’ that is observed in white matter SO. The observed 20% higher non-displaceable binding in white matter SO by Rossano et al. [30] also supports the idea that even without specific binding, [^11^C]UCB-J may behave differently in white and grey matter. However, even if there are subtle differences between the two tissue types, as long as these differences are consistent within and between groups, white matter SO could be used as a normalisation region, which seems to be the situation in the presented study group.

In the current study, SRTM2 showed good correspondence with plasma-input (NLR) derived DVR and SRTM (NLR) BP_ND_. SRTM2 BP_ND_ had an overestimation of 1.7% ± 8.8 for AD patients and an underestimation of 3.2% ± 13.4 for HCs when compared to DVR. Furthermore, SRTM2 had an overestimation of 24.0% ± 17.2 for AD patients and 16.8% ± 17.0 for HCs when compared with SRTM BP_ND_. SRTM2 is an adaptation of RPM, in which the tracer efflux rate constant k_2_’ from the reference region is fixed to a certain value by running RPM twice. In the second run, k_2_’ is fixed to the median (all voxels with BP_ND_ higher than 3) from the first run. The threshold of 3 for SRTM2 was obtained by assessing multiple values against the estimated parameter from gold standard (NLR regression model). Voxels with relatively high BP_ND_ values were used to identify the voxels that constitutes to the specific signal. These voxels were used to obtain the median k_2_’ value to perform SRTM2. Since the k_2_’ was fixed, the number of parameters were reduced from three to two, which makes the fits more reliable, since there are less parameters to estimate [26]. SRTM2 seems to perform better for [^11^C]UCB-J. SRTM2 BP_ND_ had a good correspondence with plasma input DVR, possibly due to more reliable fits. However, since SRTM2 is based on the SRTM model, the parameter estimations could still be effected by the non-identifiable k_2_’.

Among all MRTM implementations, MRTM1 BP_ND_ showed moderate correspondence with plasma input (NLR) DVR-1 with an underestimation of 25.6% ± 19 for AD patients and 20.6% ± 19 for HCs. All other MRTM images were noisy and presented quantitatively inaccurate parametric images. The lower performance of the MRTM methods could be explained by noise, as previous studies observed that noise could result in additional parameter bias in (multi) linearized methods [23, 29].

In the current study, coefficients of determination of blood-based and reference-tissue based parametric methods were validated against regional parametric estimates using NLR based models, separately for HCs and AD patients. In this regards, the performance of the blood based parametric methods (SA and Logan) was slightly better for AD patients when compared to HC subjects (Table 2). In case of reference region based parametric methods, RLogan, and MRTMo had a comparable performance between the two subject groups (Tables 2, 3) but for RPM and SRTM2 a slightly better performance for AD patients compared to HCs was observed again. Although in certain scenarios, the coefficients of determination for the comparisons of the parametric methods with the gold standard were slightly better for AD patients, there is no clear indications that any of the parametric methods actually perform better for AD patients than HCs.

Irrespective of the scan duration (90 min or 60 min), similar coefficients of determination and slopes were observed when comparing the parametric estimations for SA, RPM, and SRTM2 with the respective NLR estimations. However, the TRT slightly improved when using 90 min data for these parametric methods than when using 60 min scan data. Unfortunately, 90 min scan data was only available for HCs in the current study, therefore, this assessment was not possible to be performed on AD patients scan data. Further research on use of 90 min scan data is warranted as an improved reproducibility of the parametric estimation for these methods will be beneficial for drug intervention studies.

A recent study by Mertens et al. [12] also evaluated different parametric methods for [^11^C]UCB-J. They concluded that both SRTM2 and MRTM1 provided good parametric maps compared to RLogan. They also observed that SRTM2 BP_ND_ had the best correspondence with plasma input derived DVR. The current study also showed that BP_ND_ estimates derived from SRTM2 had the best correspondence with NLR DVR estimates. However, the performance of MRTM1 and RLogan was not similar as described by Mertens et al. [12]. One explanation could be that Mertens et al. [12] used a fixed value for the tracer efflux rate constant (*k*_2_′) when using RLogan and MRTM1 as well. So therefore, the number of parameters was limited to two, which led to less noise on the parameter estimations and eventually led to better correspondence with NLR. The current study did not use a fixed value for *k*_2_′ for RLogan and MRTM1 implementations, thereby possibly explaining the observed difference in performance.

In a previous study from our group with regional NLR analysis, TRT for NLR V_T_ and K_1_ was − 8% ± 4.3, − 2% ± 14.3 for HCs and 3% ± 8.1, 3% ± 13.6 for AD patients, respectively. TRT for Plasma-input (NLR) DVR was − 7% ± 6.2 for HCs and 7% ± 12.7 for AD patients. TRT for SRTM (NLR) derived DVR and R_1_ was − 6% ± 6.9, − 2% ± 6.3 for HCs and was − 5% ± 9.3, 2% ± 10.0 for AD patients, respectively. The HCs showed systematically lower values for the parameters V_T,_ DVR and BP_ND_ for the retest scan. The reason for finding a negative bias in the retest scan for HCs is still unclear. No technical errors (e.g. related to data acquisition or data processing), diurnal variations or changes in food intake were detected that could explain this underestimation. In the current study, a similar pattern was observed with the parametric methods too but the negative bias was slightly smaller than with conventional methods. The TRT for SA V_T_ was almost similar to the TRT of whole brain V_T_ estimated by NLR. Even though, SA K_1_ had an underestimation with reference to the NLR K_1_ estimates but the TRT for SA K_1_ was slightly better for HCs when compared to the TRT of NLR K_1_. This could be because SA approach is more robust to noise than the NLR fitting algorithm. Furthermore, whole brain TRT for SRTM2 derived BP_ND_ was similar to SRTM (NLR) derived BP_ND._ The TRT for SRTM2 R_1_ was slightly better for both groups compared to SRTM (NLR) derived R_1_. RLogan DVR had a remarkably better TRT than plasma input (NLR) DVR for both groups, however, there was an underestimation of 21.1% ± 9.3 for AD patients and 20.7% ± 8.4 for HCs when compared to plasma input (NLR) DVR and an underestimation of 9.8% ± 8.2 for AD patients and 8.8% ± 7.0 for HCs when compared to SRTM (NLR) DVR (BP_ND_ + 1), which makes RLogan not an optimal parametric method for quantification of.

In general, whole brain TRT was much better for HCs compared to AD patients for all evaluated parametric methods (Table 4). The larger variability of TRT in AD patients could be expected as the decrease in the synaptic density depends on the disease severity and the degree of atrophy that might vary from patient to patient. Although, in case of controls one would expect these differences to be minimal (no disease specific effects) and thereby a lower variability.

[^11^C]UCB-J will ultimately be used to determine synaptic density in the brain but it is also important to identify other parameters effecting the in vivo tracer kinetics such as influx and efflux of the tracer. The specific binding of the tracer is not just dependent on the availability of the receptors/targets of interest but also on the availability of the tracer. Therefore, in this study the focus was also to obtain quantitatively accurate K_1_ and R_1_ maps. As, they could help to monitor the delivery of the tracer such as influx and efflux which is of course beneficial to monitor changes in blood flow but also its effect on tracer delivery and clearance, changes that can be caused by the treatment/drug i.e. the response of a treatment, or by progression of disease.

One of the main limitations of the study was that no challenge study was performed to validate the use of white matter SO as reference region. Koole et al. [11] had validated the white matter SO as reference region for [^11^C]UCB-J PET in young healthy adults. However, the current study included relatively old HC subjects and AD patients. Since the SO is located in subcortical white matter, and white matter changes are prevalent findings in the elderly [32], re-validation of the reference region for [^11^C]UCB-J PET should be considered in further studies in older HC subjects and in patient groups. Another limitation of this study is that a few other plasma-input based voxel-level methods were not evaluated in this study, such as multilinear analysis [24], Empirical Bayesian Estimation in Graphical Analysis [33] and the Variational Bayesian inference method [34]. Similarly, for the reference region-based voxel-level methods, the reference region version of the Likelihood Estimation in Graphical Analysis [35] was not evaluated in this study. These methods could also be considered in further studies.

## Conclusions

Among the parametric approaches assessed in the current study, both SA and SRTM2 were the optimal plasma-input and reference tissue parametric methods (in comparison to the 1T2k_V_B_ model), respectively, to obtain quantitatively accurate and repeatable parametric images for [^11^C]UCB-J.

## Supplementary Information

**Additional file 1**. Coefficients of determination for V_T_estimated using Logan and SA separately for (a), (c) test and (b), (d) retest data with corresponding NLR (1T2k_V_B_) V_T_ estimates. AD: Alzheimer’s disease; HC: healthy control; LOI: line of identity.

**Additional file 2**. Scatterplots for V_T_ estimated using SA for test and retest scans, separately for (a) HCs and (b) AD patients with corresponding NLR (1T2k_V_B_) V_T_ estimates and separately color-coded for each subject. AD: Alzheimer’s disease; HC: healthy control; LOI: line of identity.

**Additional file 3**. Scatterplot for V_T_ estimated using SA with corresponding NLR (1T2k_V_B_) V_T_ estimates separately color-coded for each region.

**Additional file 4**. Scatterplot for K1 estimated using SA with corresponding NLR (1T2k_V_B_) K_1_ estimates separately color-coded for each region. 

**Additional file 5**. Coefficients of determination for RLogan, RPM, SRTM2, and MRTM1 separately for (a), (c), (e), (g) test and (b), (d), (f), (h) retest data with corresponding NLR (1T2k_V_B_) estimates. AD: Alzheimer’s disease; HC: healthy control; LOI: line of identity; BP_ND_: binding potential; DVR: distribution volume ratio; NLR: non-linear regression; RLogan: reference Logan; RPM: receptor parametric mapping; SRTM2: simplified reference tissue model 2; MRTM1: multilinear reference tissue model 1.

**Additional file 6**. Scatterplots for BP_ND_ estimated using RPM for test and retest scans, separately for (a) HCs and (b) AD patients with corresponding NLR (1T2k_V_B_) DVR-1 estimates and separately color-coded for each subject. AD: Alzheimer’s disease; HC: healthy control; LOI: line of identity.

**Additional file 7**. Scatterplots for BP_ND_ estimated using SRTM2 for test and retest scans, separately for (a) HCs and (b) AD patients with corresponding NLR (1T2k_V_B_) DVR-1 estimates and separately color-coded for each subject. AD: Alzheimer’s disease; HC: healthy control; LOI: line of identity.

**Additional file 8**. Scatterplot for BP_ND_ estimated using SRTM2 with corresponding NLR (1T2k_V_B_) DVR-1 estimates separately color-coded for each region.

**Additional file 9**. Scatterplot for R_1_ estimated using SRTM2 with corresponding NLR (SRTM) R_1_ estimates separately color-coded for each region. 

**Additional file 10**. TRT repeatability voxel-wise image (average and SD) for AD patients separately for SA V_T_, RPM BP_ND_ and SRTM2 BP_ND_.

**Additional file 11**. TRT repeatability voxel-wise image (average and SD) for HCs separately for SA V_T_, RPM BP_ND_ and SRTM2 BP_ND_.

**Additional file 12**. The % bias (mean + SD) estimated for the parametric methods of interest against corresponding 1T2k_V_B_ estimates using 60 minutes data.

**Additional file 13**. Coefficients of determination (r^2^) and slopes of parametric [^11^C]UCB-J V_T_, K_1_ and BP_ND_ against corresponding 1T2k_V_B_ estimates separately for all subjects. All the Hammers ROIs were included for this analysis. Regional parametric values from both test and retest scans were pulled together for these comparisons.

**Additional file 14**. Coefficients of determination (r^2^) and slopes of parametric [^11^C]UCB-J BP_ND_ and R_1_ against corresponding SRTM estimates separately for all subjects. All the Hammers ROIs were included for this analysis. Regional parametric values from both test and retest scans were pulled together for these comparisons.

**Additional file 15**. The % bias (mean + SD) estimated for the parametric methods of interest against corresponding SRTM estimates using 60 minutes data.

**Additional file 16**. TRT (%) values estimated for specific brain regions (grey matter) are presented for SA V_T_ and K_1_.

**Additional file 17**. TRT (%) values estimated for specific brain regions (grey matter) are presented for SRTM2 BP_ND_ and R_1_.

**Additional file 18**. TRT (%) values estimated for specific brain regions (grey matter) are presented for RPM BP_ND_ and R_1_.

**Additional file 19**. Coefficients of determination (r^2^) and slopes of parametric [^11^C]UCB-J V_T_, K_1_ and BP_ND_ against corresponding 1T2k_V_B_ estimates for HCs using 90 minutes data. All the Hammers ROIs were included for this analysis.

**Additional file 20**. Coefficients of determination (r^2^) and slopes of parametric [^11^C]UCB-J BP_ND_ and R_1_ against corresponding SRTM estimates using 90 minutes data. All the Hammers ROIs were included for this analysis.

**Additional file 21**. TRT (%) values estimated for whole brain (grey matter) are presented for each parametric method using 90 minutes data.

## Data Availability

The datasets used and/or analysed during the current study are available from the corresponding author on reasonable request.

## Abbreviations

2T4k_V_B_: Reversible 2 tissue compartment model with blood volume correction
[^11^C]UCB-J: (R)-1-((3-([^11^C]methyl)pyridin-4-yl)methyl)-4-(3,4,5-trifluorophenyl)pyrrolidin-2-one
AD: Azheimer’s disease
Amsterdam UMC: Amsterdam University Medical Center
BP_ND_: Binding potential
CT: Computed tomography
DVR: Distribution volume ratio
HC: Healthy control
K_1_: Rate of influx of radioligand from blood to tissue
Logan: Plasma input Logan
MMSE: Mini-Mental State Examination
MREC: Medical Review and Ethics Committee
MRTM: Multilinear reference tissue model
NFTs: Neurofibrillary tangles
NIA-AA: National Institute on Aging—Alzheimer’s Association
NLR: Nonlinear regression
OSEM: Ordered-subsets-expectation–maximization
PET: Positron emission tomography
Rlogan: Reference Logan
ROI: Regions of interest
RPM: Receptor parametric mapping
SA: Spectral analysis
SO: Semi-ovale
SRTM2: Simplified reference tissue model 2
SUVr: Standardized uptake value ratio
SV2A: Synaptic vesicle protein 2A
TAC: Time activity curve
TOF: Time of flight
TRT: Test–retest repeatability
V_T_: Total volume of distribution
