# Supplementary figures and images for: Validation and test–retest repeatability performance of parametric methods for [11C]UCB-J PET
Source: EJNMMI Res. 2022 Jan 24;12:3. doi: 10.1186/s13550-021-00874-8 (PMC8786991; doi:10.1186/s13550-021-00874-8)

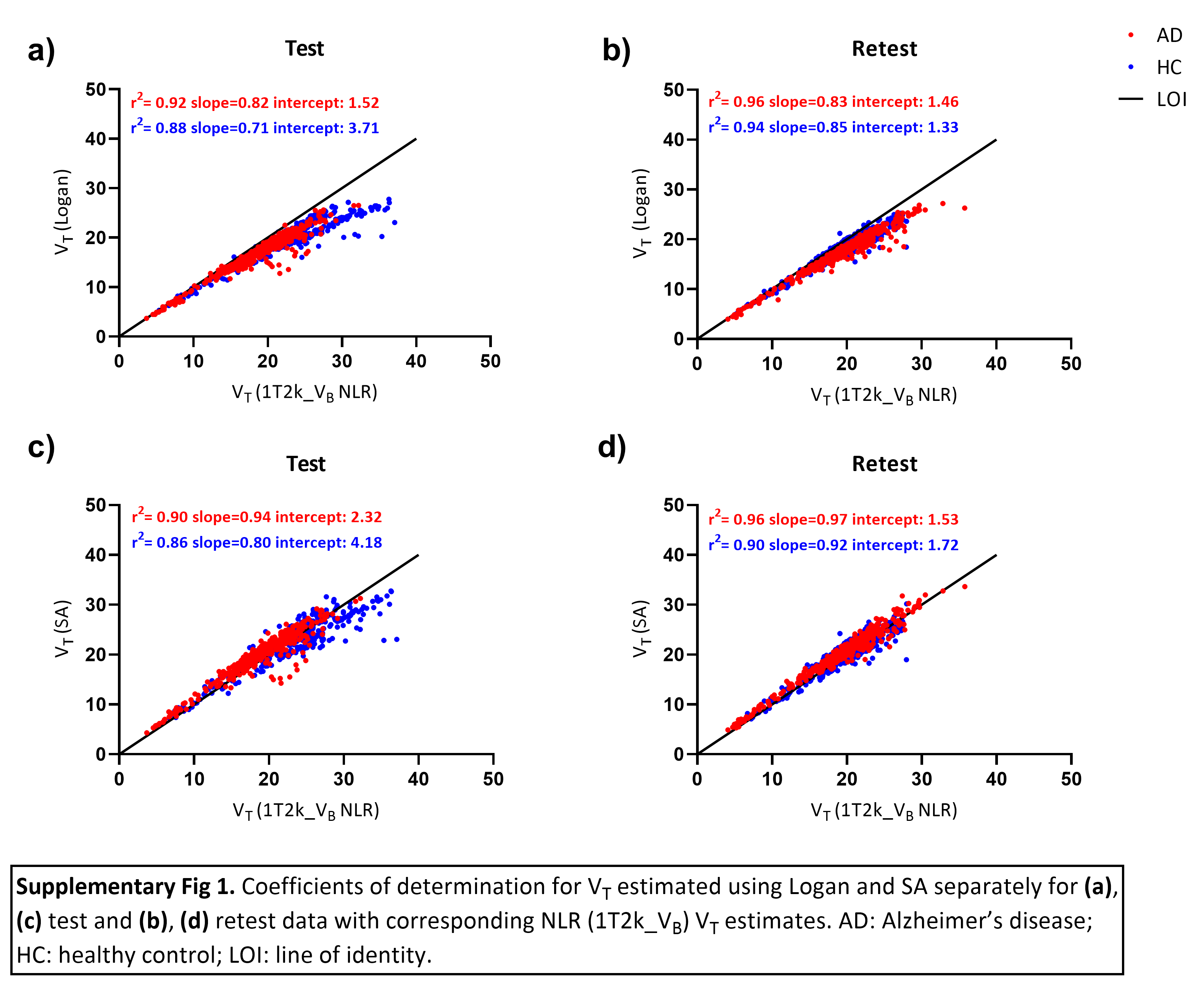

Supplement: Supplementary file 1 — Additional file 1. Coefficients of determination for VTestimated using Logan and SA separately for (a), (c) test and (b), (d) retest data with corresponding NLR (1T2k_VB) VT estimates. AD: Alzheimer’s disease; HC: healthy control; LOI: line of identity. [file 13550_2021_874_MOESM1_ESM.png]

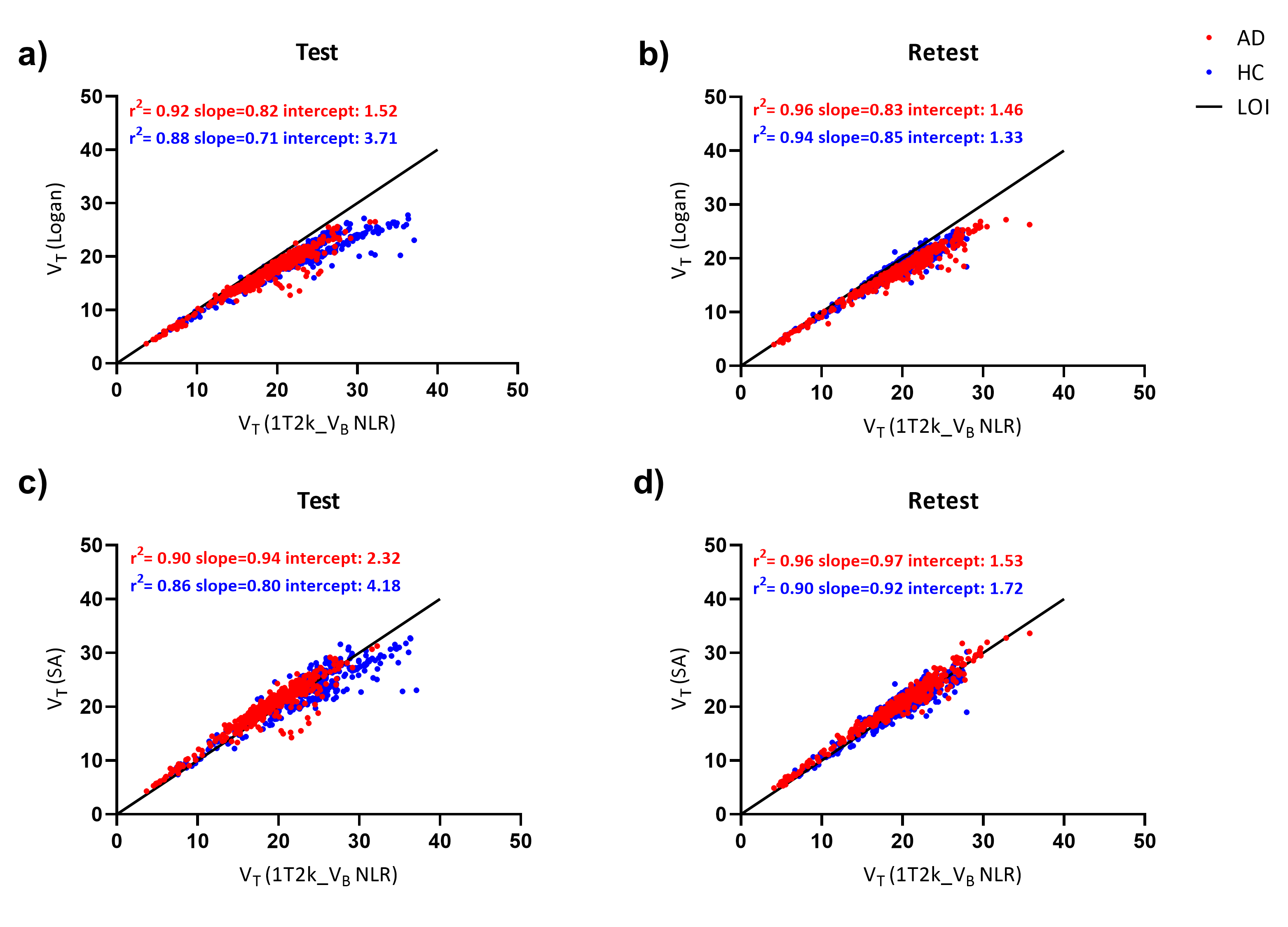

Supplement: Supplementary file 2 — Additional file 2. Scatterplots for VT estimated using SA for test and retest scans, separately for (a) HCs and (b) AD patients with corresponding NLR (1T2k_VB) VT estimates and separately color-coded for each subject. AD: Alzheimer’s disease; HC: healthy control; LOI: line of identity. [file 13550_2021_874_MOESM2_ESM.png]

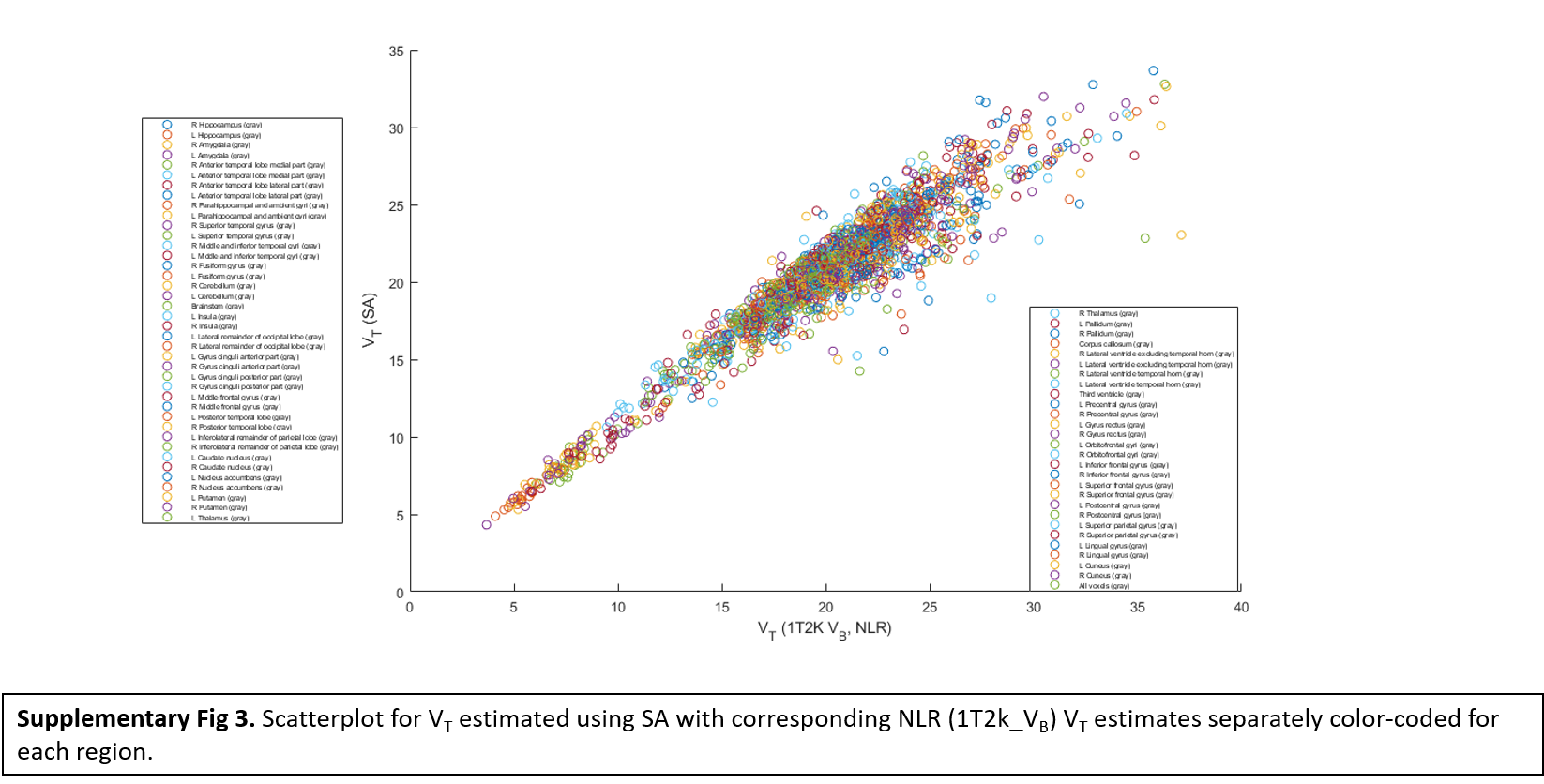

Supplement: Supplementary file 3 — Additional file 3. Scatterplot for VT estimated using SA with corresponding NLR (1T2k_VB) VT estimates separately color-coded for each region. [file 13550_2021_874_MOESM3_ESM.png]

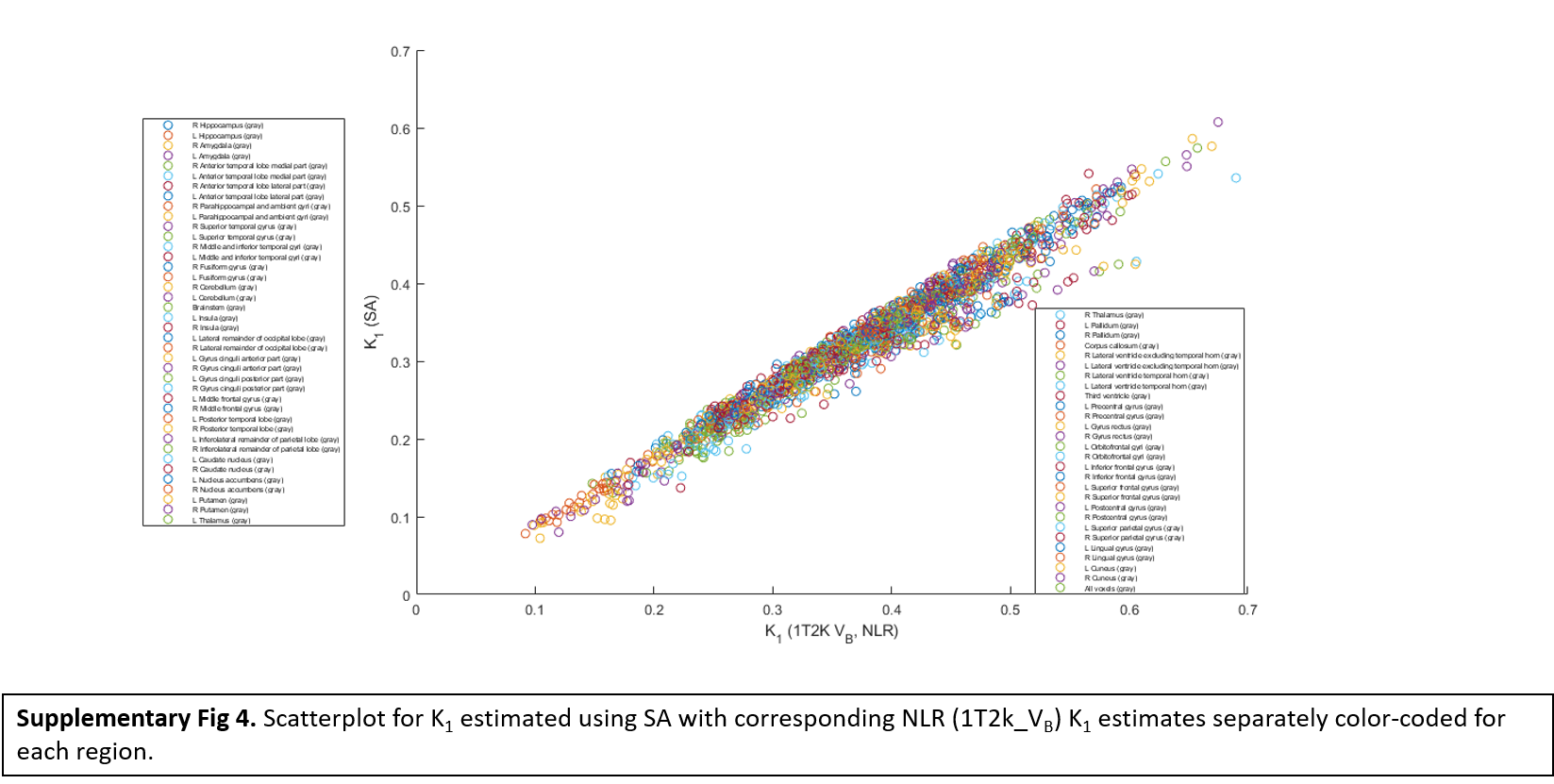

Supplement: Supplementary file 4 — Additional file 4. Scatterplot for K1 estimated using SA with corresponding NLR (1T2k_VB) K1 estimates separately color-coded for each region. [file 13550_2021_874_MOESM4_ESM.png]

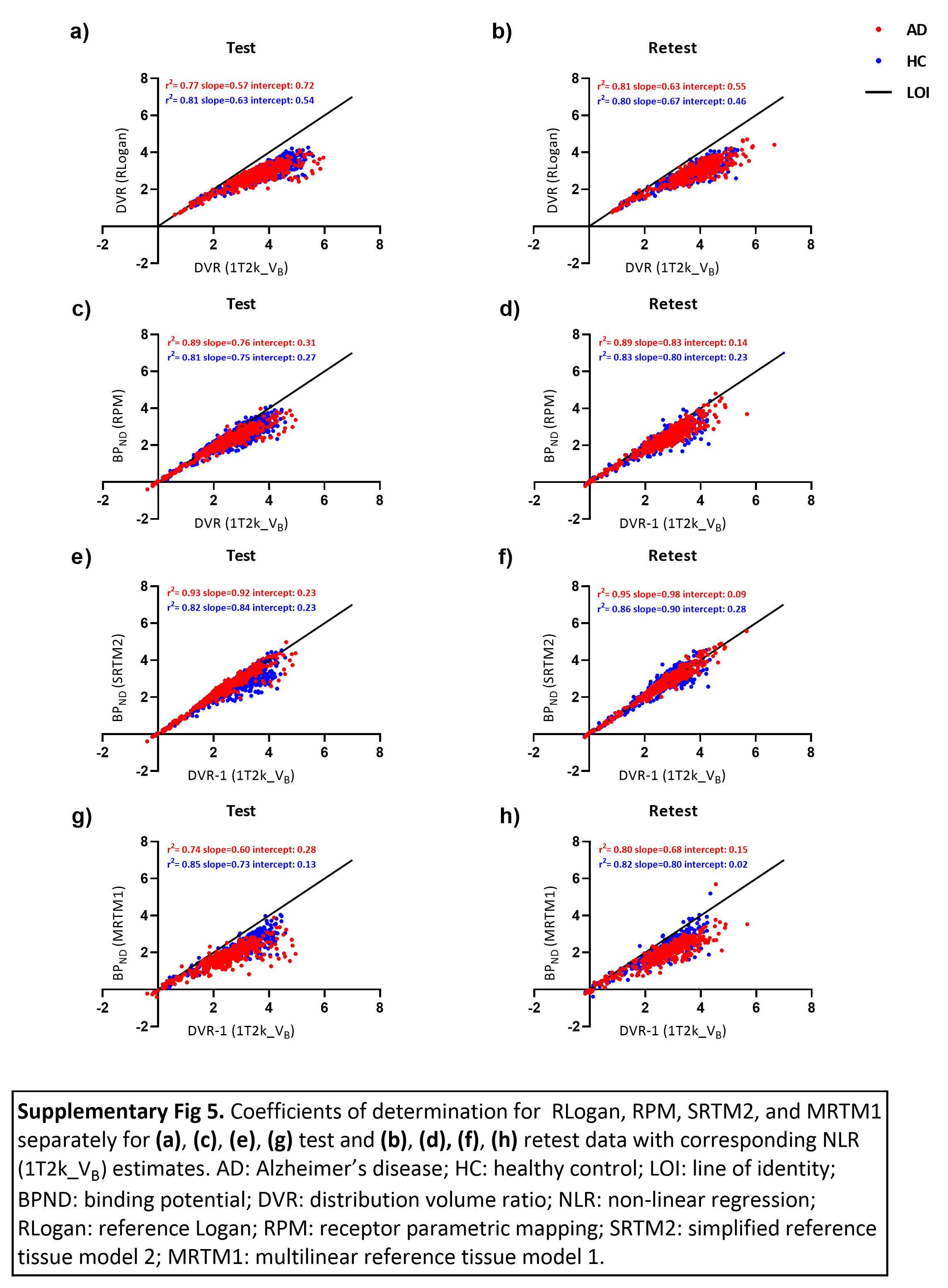

Supplement: Supplementary file 5 — Additional file 5. Coefficients of determination for RLogan, RPM, SRTM2, and MRTM1 separately for (a), (c), (e), (g) test and (b), (d), (f), (h) retest data with corresponding NLR (1T2k_VB) estimates. AD: Alzheimer’s disease; HC: healthy control; LOI: line of identity; BPND: binding potential; DVR: distribution volume ratio; NLR: non-linear regression; RLogan: reference Logan; RPM: receptor parametric mapping; SRTM2: simplified reference tissue model 2; MRTM1: multilinear reference tissue model 1. [file 13550_2021_874_MOESM5_ESM.png]

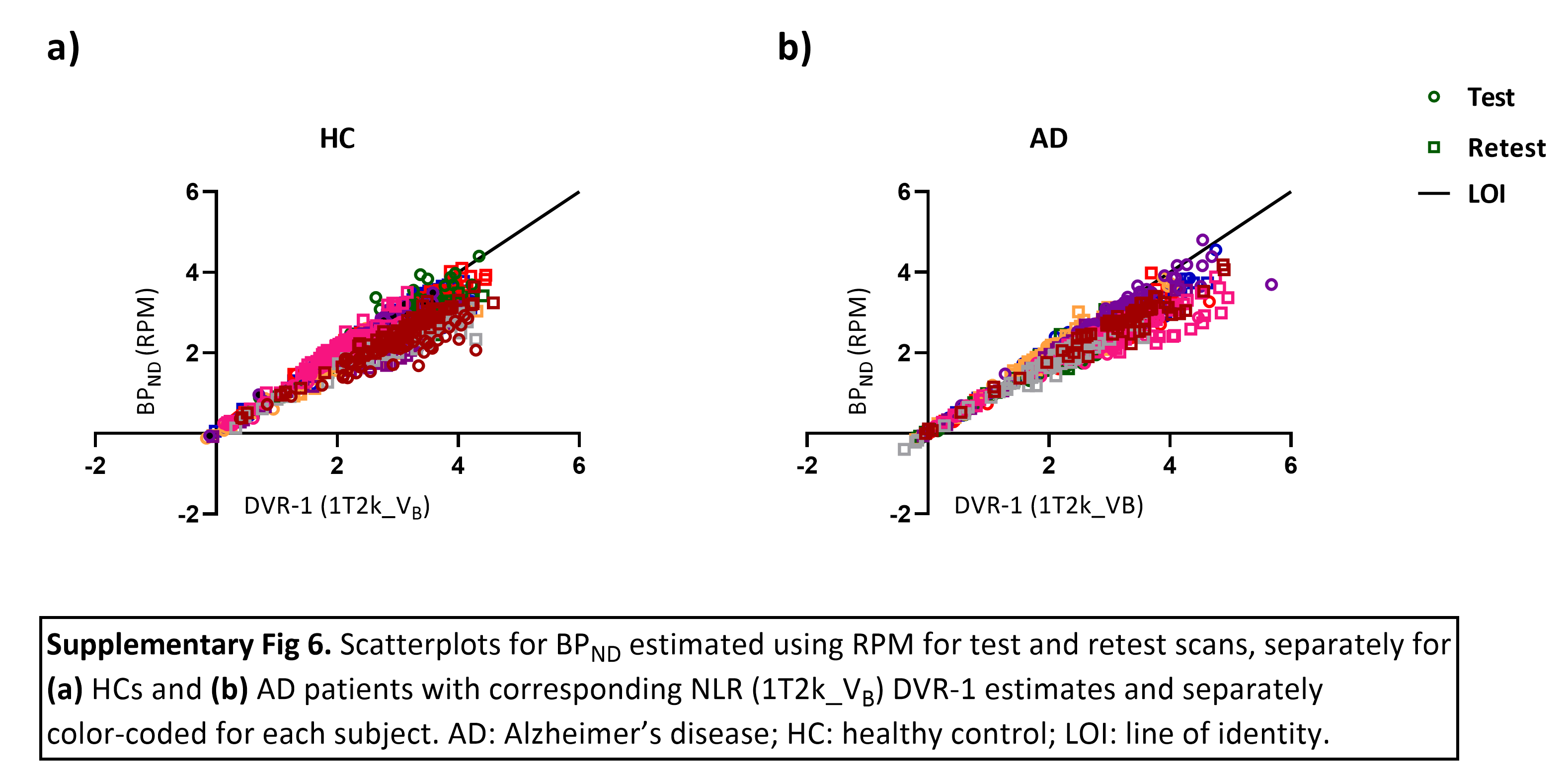

Supplement: Supplementary file 6 — Additional file 6. Scatterplots for BPND estimated using RPM for test and retest scans, separately for (a) HCs and (b) AD patients with corresponding NLR (1T2k_VB) DVR-1 estimates and separately color-coded for each subject. AD: Alzheimer’s disease; HC: healthy control; LOI: line of identity. [file 13550_2021_874_MOESM6_ESM.png]

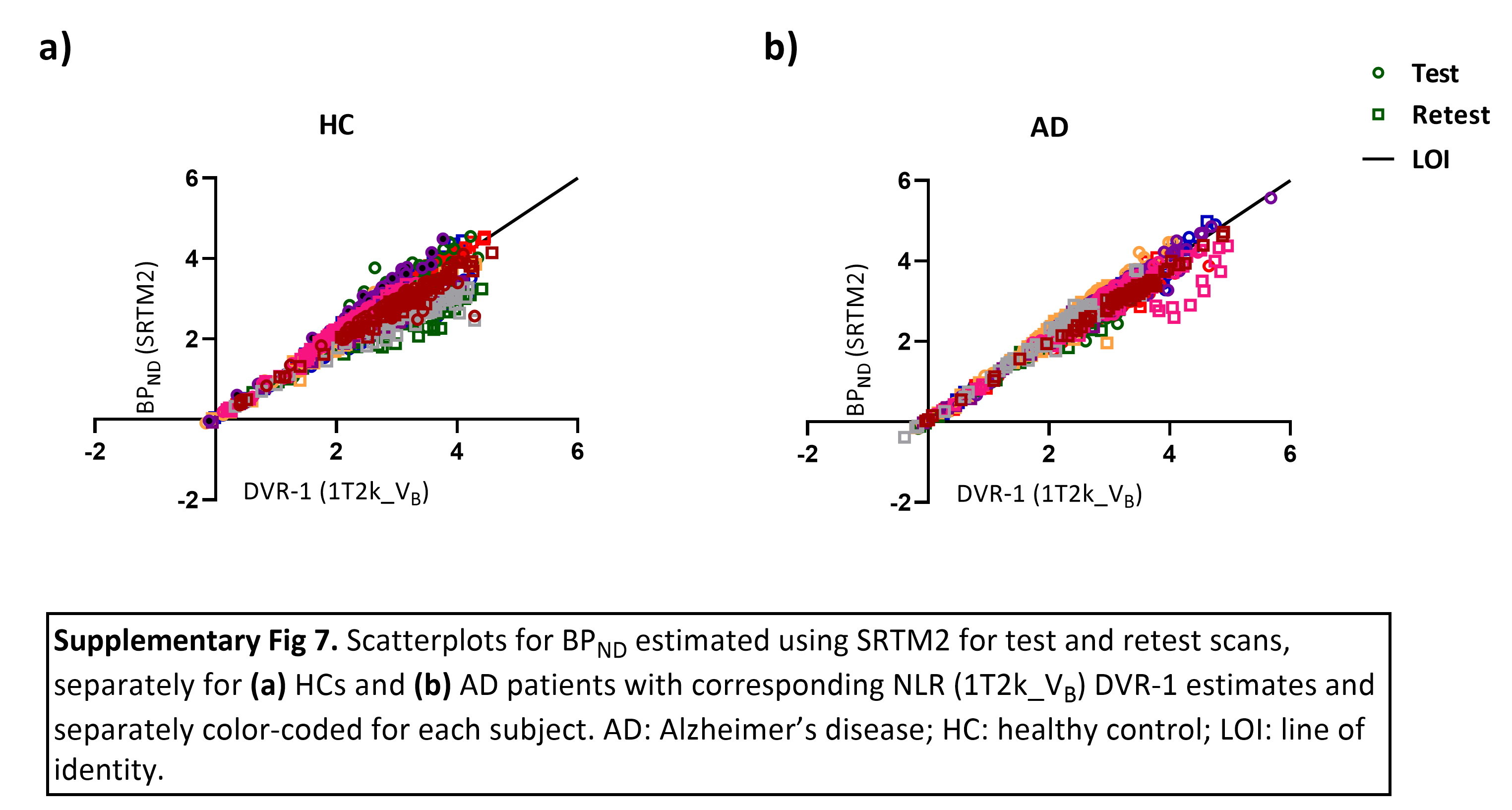

Supplement: Supplementary file 7 — Additional file 7. Scatterplots for BPND estimated using SRTM2 for test and retest scans, separately for (a) HCs and (b) AD patients with corresponding NLR (1T2k_VB) DVR-1 estimates and separately color-coded for each subject. AD: Alzheimer’s disease; HC: healthy control; LOI: line of identity. [file 13550_2021_874_MOESM7_ESM.png]

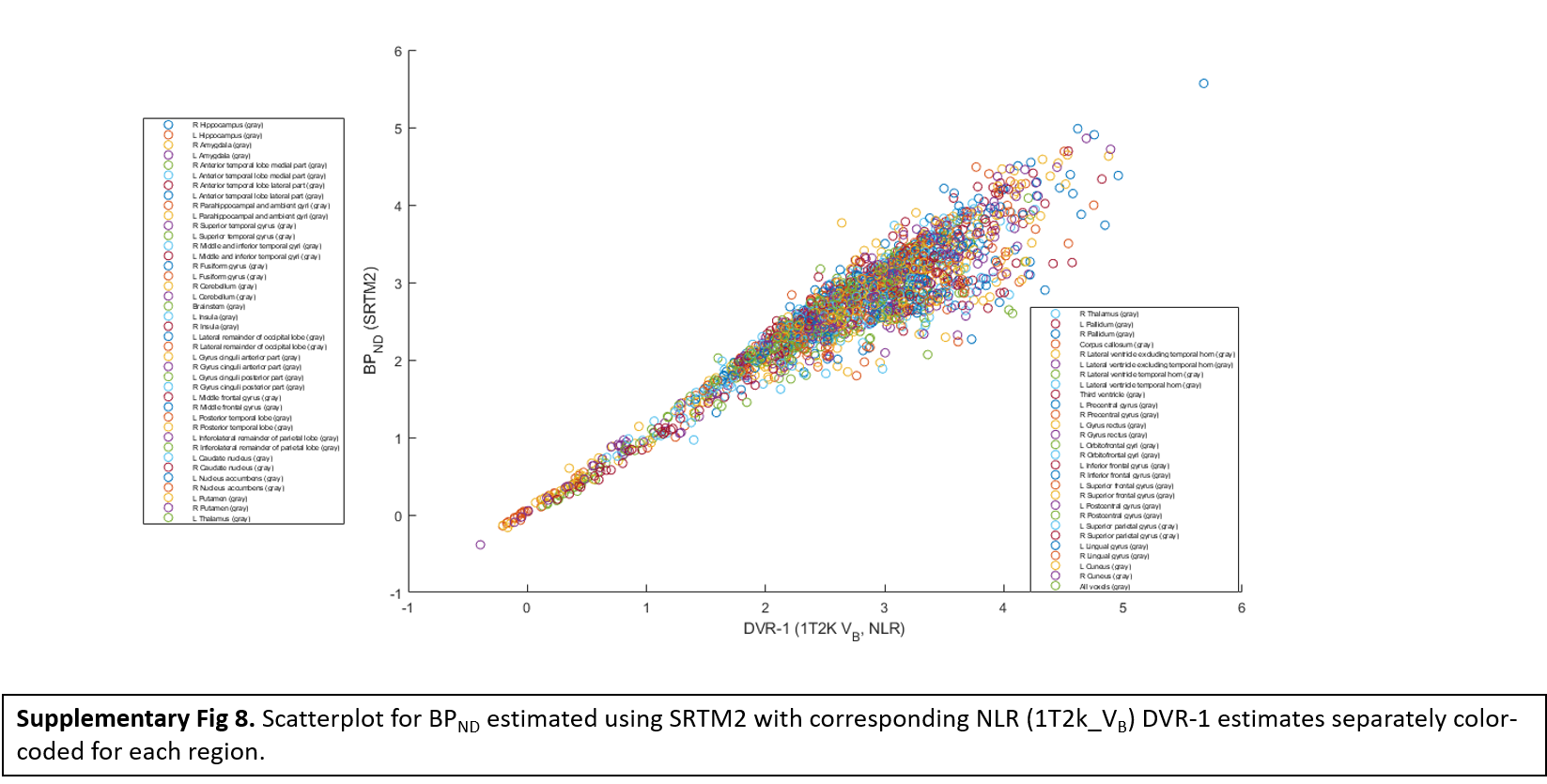

Supplement: Supplementary file 8 — Additional file 8. Scatterplot for BPND estimated using SRTM2 with corresponding NLR (1T2k_VB) DVR-1 estimates separately color-coded for each region. [file 13550_2021_874_MOESM8_ESM.png]

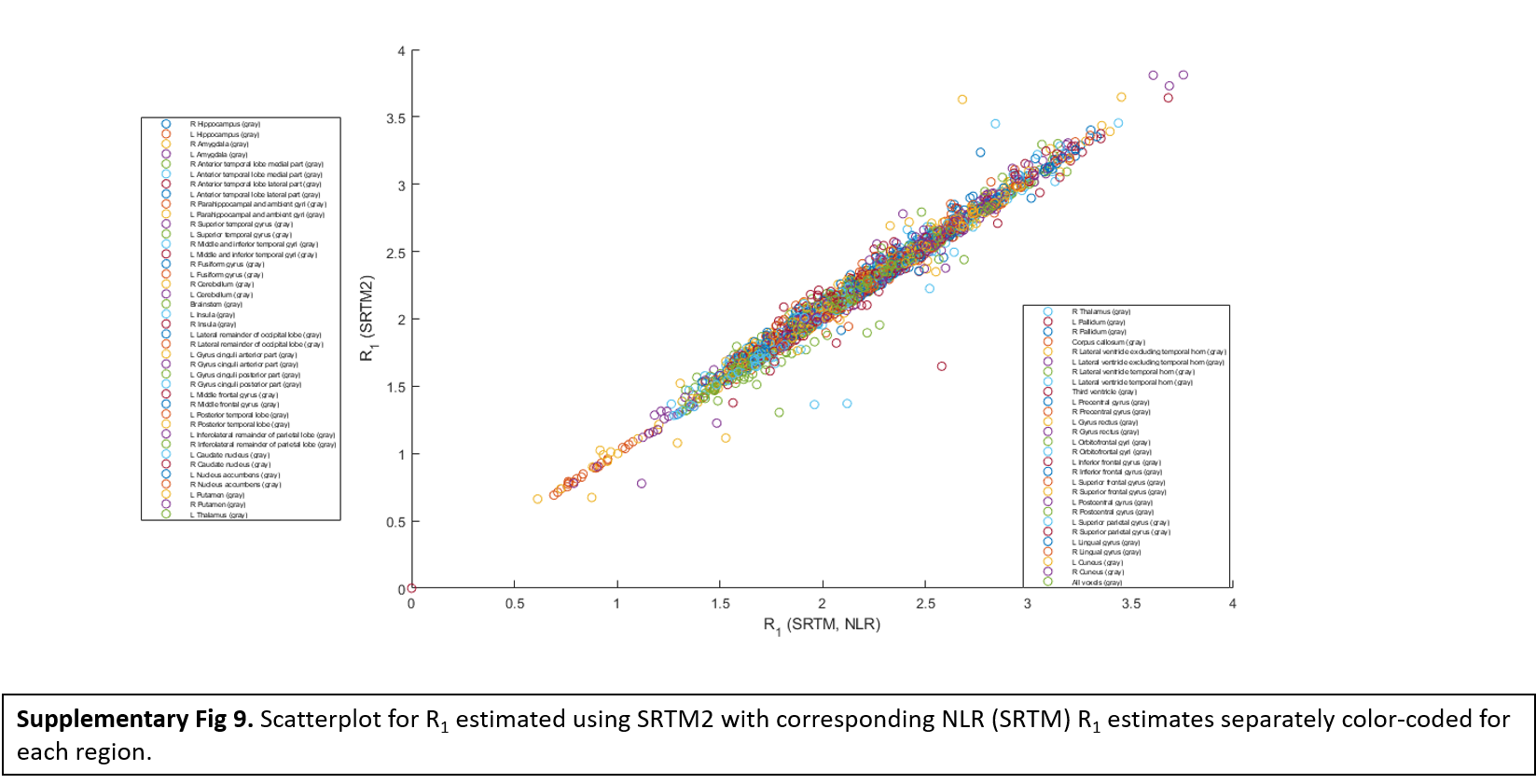

Supplement: Supplementary file 9 — Additional file 9. Scatterplot for R1 estimated using SRTM2 with corresponding NLR (SRTM) R1 estimates separately color-coded for each region. [file 13550_2021_874_MOESM9_ESM.png]

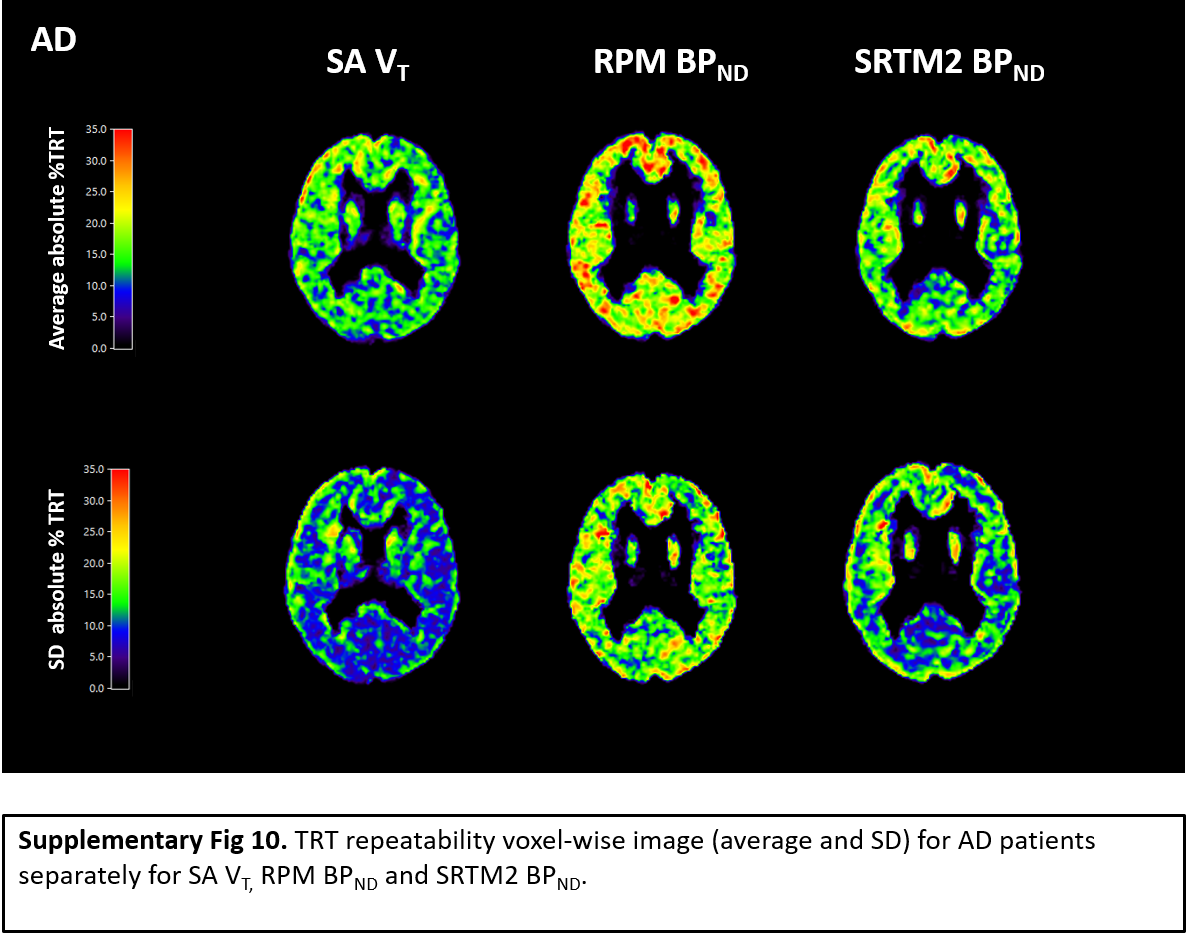

Supplement: Supplementary file 10 — Additional file 10. TRT repeatability voxel-wise image (average and SD) for AD patients separately for SA VT, RPM BPND and SRTM2 BPND. [file 13550_2021_874_MOESM10_ESM.png]

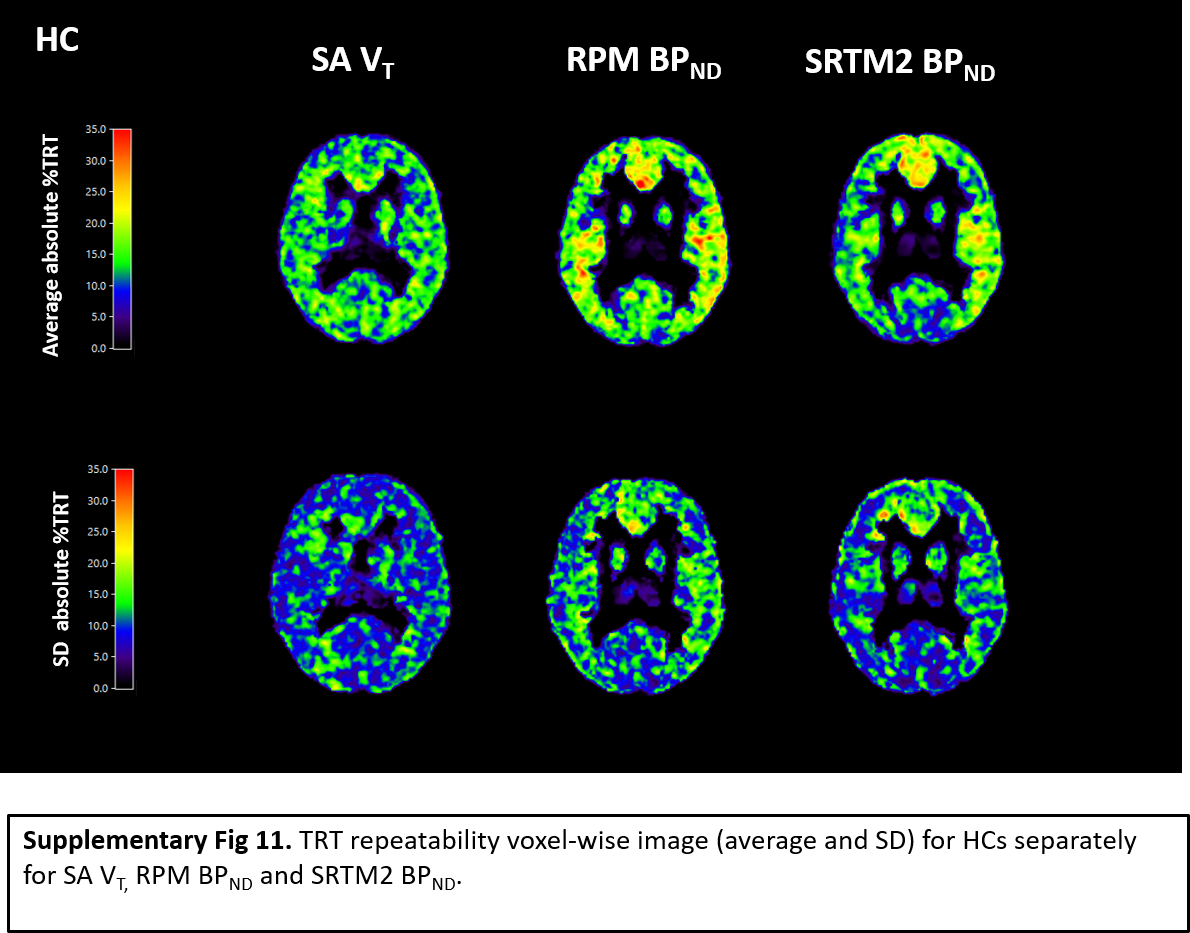

Supplement: Supplementary file 11 — Additional file 11. TRT repeatability voxel-wise image (average and SD) for HCs separately for SA VT, RPM BPND and SRTM2 BPND. [file 13550_2021_874_MOESM11_ESM.png]
