# Supplementary material for: Validation and test–retest repeatability performance of parametric methods for [11C]UCB-J PET
Source: EJNMMI Res. 2022 Jan 24;12:3. doi: 10.1186/s13550-021-00874-8 (PMC8786991; doi:10.1186/s13550-021-00874-8)
Supplement: Supplementary file 12 — Additional file 12. The % bias (mean + SD) estimated for the parametric methods of interest against corresponding 1T2k_VB estimates using 60 minutes data. [file 13550_2021_874_MOESM12_ESM.docx]

**Supplementary Table 1.** The % bias (mean + SD) estimated for the parametric methods of interest against corresponding 1T2k_V_B_ estimates using 60 minutes data.

|  |  | |  | |
| --- | --- | --- | --- | --- |
|  | **HC** | | **AD** | |
|  | **Mean** | **SD** | **Mean** | **SD** |
| **SA V_T_** | 0.5 | 7.6 | 5.7 | 6.7 |
| **SA K_1_** | -13.2 | 4.9 | -14.1 | 4.7 |
| **RPM BP_ND_** | -12.2 | 12.9 | -11.0 | 10.8 |
| **SRTM2 BP_ND_** | -3.2 | 13.4 | 1.7 | 8.8 |
