# Supplementary material for: Validation and test–retest repeatability performance of parametric methods for [11C]UCB-J PET
Source: EJNMMI Res. 2022 Jan 24;12:3. doi: 10.1186/s13550-021-00874-8 (PMC8786991; doi:10.1186/s13550-021-00874-8)
Supplement: Supplementary file 13 — Additional file 13. Coefficients of determination (r2) and slopes of parametric [11C]UCB-J VT, K1 and BPND against corresponding 1T2k_VB estimates separately for all subjects. All the Hammers ROIs were included for this analysis. Regional parametric values from both test and retest scans were pulled together for these comparisons. [file 13550_2021_874_MOESM13_ESM.docx]

|  | **SA V_T_** | | | **SA K_1_** | | | **SRTM2 BP_ND_** | | | **RPM BP_ND_** | | |
| --- | --- | --- | --- | --- | --- | --- | --- | --- | --- | --- | --- | --- |
|  | | ***r*^2^** | ***Slope*** | | ***r*^2^** | **Slope** | | ***r*^2^** | **Slope** | | ***r*^2^** | **Slope** |
| **HC 1** | | 0.96 | 0.93 | | 0.97 | 0.84 | | 0.93 | 1.01 | | 0.92 | 0.87 |
| **HC 2** | | 0.96 | 0.98 | | 0.99 | 0.90 | | 0.99 | 1.01 | | 0.94 | 0.85 |
| **HC 3** | | 0.89 | 0.96 | | 0.97 | 0.90 | | 0.86 | 0.72 | | 0.93 | 0.77 |
| **HC 4** | | 0.95 | 0.81 | | 0.84 | 0.71 | | 0.94 | 0.92 | | 0.91 | 0.69 |
| **HC 5** | | 0.98 | 1.00 | | 0.98 | 0.88 | | 0.98 | 0.81 | | 0.82 | 0.71 |
| **HC 6** | | 0.92 | 0.98 | | 0.97 | 0.92 | | 0.97 | 1.05 | | 0.91 | 0.93 |
| **HC 7** | | 0.85 | 0.78 | | 0.99 | 0.73 | | 0.92 | 0.79 | | 0.90 | 0.72 |
| **HC 8** | | 0.90 | 0.97 | | 0.99 | 0.82 | | 0.93 | 0.87 | | 0.82 | 0.70 |
| **AD 1** | | 0.96 | 0.97 | | 0.94 | 0.88 | | 0.98 | 1.02 | | 0.94 | 0.81 |
| **AD 2** | | 0.97 | 0.98 | | 0.99 | 0.87 | | 0.95 | 0.90 | | 0.95 | 0.78 |
| **AD 3** | | 0.96 | 0.91 | | 0.96 | 0.85 | | 0.97 | 0.94 | | 0.93 | 0.82 |
| **AD 4** | | 0.97 | 1.01 | | 0.99 | 0.89 | | 0.96 | 1.06 | | 0.95 | 0.91 |
| **AD 5** | | 0.81 | 0.78 | | 0.98 | 0.89 | | 0.87 | 0.82 | | 0.87 | 0.63 |
| **AD 6** | | 0.97 | 0.98 | | 0.97 | 0.86 | | 0.97 | 1.01 | | 0.92 | 0.87 |
| **AD 7** | | 0.97 | 0.97 | | 0.99 | 0.87 | | 0.97 | 0.92 | | 0.95 | 0.81 |

**Supplementary Table 2:** Coefficients of determination (r^2^) and slopes of parametric [^11^C]UCB-J V_T_, K_1_ and BP_ND_ against corresponding 1T2k_V_B_ estimates separately for all subjects. All the Hammers ROIs were included for this analysis. Regional parametric values from both test and retest scans were pulled together for these comparisons.
