# Supplementary material for: Validation and test–retest repeatability performance of parametric methods for [11C]UCB-J PET
Source: EJNMMI Res. 2022 Jan 24;12:3. doi: 10.1186/s13550-021-00874-8 (PMC8786991; doi:10.1186/s13550-021-00874-8)
Supplement: Supplementary file 14 — Additional file 14. Coefficients of determination (r2) and slopes of parametric [11C]UCB-J BPND and R1 against corresponding SRTM estimates separately for all subjects. All the Hammers ROIs were included for this analysis. Regional parametric values from both test and retest scans were pulled together for these comparisons. [file 13550_2021_874_MOESM14_ESM.docx]

**Supplementary Table 3:** Coefficients of determination (r^2^) and slopes of parametric [^11^C]UCB-J BP_ND_ and R_1_ against corresponding SRTM estimates separately for all subjects. All the Hammers ROIs were included for this analysis. Regional parametric values from both test and retest scans were pulled together for these comparisons.

|  | **SRTM2 BP_ND_** | | | **SRTM2 R_1_** | | | **RPM BP_ND_** | | | **RPM R_1_** | | |
| --- | --- | --- | --- | --- | --- | --- | --- | --- | --- | --- | --- | --- |
|  | | ***r*^2^** | ***Slope*** | | ***r*^2^** | **Slope** | | ***r*^2^** | **Slope** | | ***r*^2^** | **Slope** |
| **HC 1** | | 0.88 | 1.15 | | 0.99 | 1.01 | | 0.96 | 1.03 | | 1.00 | 1.01 |
| **HC 2** | | 0.91 | 1.16 | | 1.00 | 1.01 | | 0.97 | 1.02 | | 1.00 | 0.99 |
| **HC 3** | | 0.77 | 0.95 | | 0.94 | 1.05 | | 0.96 | 0.95 | | 0.95 | 1.03 |
| **HC 4** | | 0.93 | 1.23 | | 0.99 | 1.02 | | 0.96 | 0.94 | | 0.99 | 1.00 |
| **HC 5** | | 0.78 | 1.16 | | 0.98 | 1.01 | | 0.83 | 0.99 | | 1.00 | 1.00 |
| **HC 6** | | 0.87 | 1.25 | | 0.93 | 0.94 | | 0.94 | 1.08 | | 0.98 | 0.98 |
| **HC 7** | | 0.96 | 1.13 | | 0.99 | 1.06 | | 0.97 | 1.08 | | 0.99 | 1.02 |
| **HC 8** | | 0.85 | 1.11 | | 0.99 | 1.02 | | 0.87 | 0.96 | | 1.00 | 1.00 |
| **AD 1** | | 0.87 | 1.14 | | 0.98 | 1.03 | | 0.97 | 0.99 | | 0.99 | 0.99 |
| **AD 2** | | 0.92 | 1.15 | | 0.97 | 0.99 | | 0.98 | 1.01 | | 1.00 | 1.00 |
| **AD 3** | | 0.95 | 1.18 | | 0.95 | 1.01 | | 0.98 | 1.03 | | 0.96 | 1.00 |
| **AD 4** | | 0.94 | 1.27 | | 0.94 | 0.97 | | 0.99 | 1.06 | | 0.97 | 0.98 |
| **AD 5** | | 0.87 | 1.28 | | 0.98 | 1.02 | | 0.98 | 1.06 | | 1.00 | 1.01 |
| **AD 6** | | 0.92 | 1.16 | | 0.97 | 0.99 | | 0.92 | 0.96 | | 0.98 | 0.99 |
| **AD 7** | | 0.94 | 1.18 | | 0.99 | 1.00 | | 0.96 | 1.07 | | 1.00 | 0.99 |
