# Supplementary material for: Validation and test–retest repeatability performance of parametric methods for [11C]UCB-J PET
Source: EJNMMI Res. 2022 Jan 24;12:3. doi: 10.1186/s13550-021-00874-8 (PMC8786991; doi:10.1186/s13550-021-00874-8)
Supplement: Supplementary file 15 — Additional file 15. The % bias (mean + SD) estimated for the parametric methods of interest against corresponding SRTM estimates using 60 minutes data. [file 13550_2021_874_MOESM15_ESM.docx]

|  |  | |  | |
| --- | --- | --- | --- | --- |
|  | **HC** | | **AD** | |
|  | **Mean** | **SD** | **Mean** | **SD** |
| **RPM BP_ND_** | 4.3 | 9.5 | 5.8 | 7.3 |
| **RPM R_1_** | -1.0 | 2.1 | -1.7 | 3.0 |
| **SRTM2 BP_ND_** | 16.8 | 17.0 | 24.0 | 17.2 |
| **SRTM2 R_1_** | 1.9 | 3.1 | 2.7 | 4.4 |

**Supplementary Table 4.** The % bias (mean + SD) estimated for the parametric methods of interest against corresponding SRTM estimates using 60 minutes data.
