# Supplementary material for: Validation and test–retest repeatability performance of parametric methods for [11C]UCB-J PET
Source: EJNMMI Res. 2022 Jan 24;12:3. doi: 10.1186/s13550-021-00874-8 (PMC8786991; doi:10.1186/s13550-021-00874-8)
Supplement: Supplementary file 16 — Additional file 16. TRT (%) values estimated for specific brain regions (grey matter) are presented for SA VT and K1. [file 13550_2021_874_MOESM16_ESM.docx]

**Supplementary Table 5:** TRT (%) values estimated for specific brain regions (grey matter) are presented for SA V_T_ and K_1_.

|  | **SA V_T_** | | | | **SA K_1_** | | | |
| --- | --- | --- | --- | --- | --- | --- | --- | --- |
|  | **HC** | | **AD** | | **HC** | | **AD** | |
|  | **TRT%** | **SD** | **TRT%** | **SD** | **TRT%** | **SD** | **TRT%** | **SD** |
| **Medial Temporal Lobe** | -8 | -7 | 4 | 7 | 1 | 13 | 7 | 12 |
| **Frontal Cortex** | -7 | 5 | 2 | 9 | 1 | 11 | 7 | 12 |
| **Parietal Cortex** | -8 | 9 | 0 | 7 | 2 | 12 | 6 | 11 |
| **Temporal Cortex** | -10 | 5 | 3 | 7 | 2 | 11 | 5 | 11 |
| **Occipital Cortex** | -8 | 7 | 1 | 7 | 3 | 12 | 6 | 14 |
| **Anterior Cingulate Cortex** | -10 | 7 | 0 | 10 | 1 | 13 | 1 | 18 |
| **Posterior Cingulate Cortex** | -13 | 10 | 1 | 11 | 2 | 14 | 6 | 13 |
| **Thalamus** | -6 | 9 | 5 | 13 | 0 | 14 | 8 | 11 |
| **Putamen** | -9 | 5 | 2 | 8 | 2 | 13 | 8 | 14 |
| **Caudate Nucleus** | -7 | 8 | 2 | 13 | 1 | 11 | 11 | 13 |
| **Hippocampus** | -9 | 9 | 2 | 7 | -1 | 13 | 8 | 13 |
| **Cerebellum** | -11 | 8 | 3 | 10 | 0 | 13 | 6 | 13 |
| **Brainstem** | -7 | 7 | 2 | 14 | -2 | 15 | 4 | 14 |
