# Supplementary material for: Validation and test–retest repeatability performance of parametric methods for [11C]UCB-J PET
Source: EJNMMI Res. 2022 Jan 24;12:3. doi: 10.1186/s13550-021-00874-8 (PMC8786991; doi:10.1186/s13550-021-00874-8)
Supplement: Supplementary file 17 — Additional file 17. TRT (%) values estimated for specific brain regions (grey matter) are presented for SRTM2 BPND and R1. [file 13550_2021_874_MOESM17_ESM.docx]

**Supplementary Table 6:** TRT (%) values estimated for specific brain regions (grey matter) are presented for SRTM2 BP_ND_ and R_1_.

|  | **SRTM2 BP_ND_** | | | | **SRTM2 R_1_** | | | |
| --- | --- | --- | --- | --- | --- | --- | --- | --- |
|  | **HC** | | **AD** | | **HC** | | **AD** | |
|  | **TRT%** | **SD** | **TRT%** | **SD** | **TRT%** | **SD** | **TRT%** | **SD** |
| **Medial Temporal Lobe** | 0 | 21 | 12 | 18 | -2 | 9 | 4 | 9 |
| **Frontal Cortex** | -2 | 14 | 8 | 13 | -1 | 7 | 3 | 9 |
| **Parietal Cortex** | -3 | 12 | 7 | 11 | -1 | 7 | 3 | 9 |
| **Temporal Cortex** | -1 | 18 | 9 | 14 | -1 | 7 | 2 | 9 |
| **Occipital Cortex** | -3 | 9 | 9 | 14 | 0 | 7 | 3 | 8 |
| **Anterior Cingulate Cortex** | 0 | 24 | 2 | 14 | -2 | 7 | -1 | 5 |
| **Posterior Cingulate Cortex** | -4 | 13 | 3 | 5 | 0 | 6 | 3 | 9 |
| **Thalamus** | -3 | 5 | 16 | 18 | -2 | 7 | 3 | 10 |
| **Putamen** | -1 | 16 | 8 | 12 | -1 | 6 | 4 | 8 |
| **Caudate Nucleus** | -0 | 15 | 10 | 18 | -1 | 6 | 6 | 9 |
| **Hippocampus** | -4 | 19 | 10 | 18 | -4 | 8 | 4 | 7 |
| **Cerebellum** | -5 | 7 | 28 | 15 | -2 | 6 | 3 | 9 |
| **Brainstem** | -18 | 22 | 9 | 28 | -2 | 6 | 3 | 7 |
