# Supplementary material for: Validation and test–retest repeatability performance of parametric methods for [11C]UCB-J PET
Source: EJNMMI Res. 2022 Jan 24;12:3. doi: 10.1186/s13550-021-00874-8 (PMC8786991; doi:10.1186/s13550-021-00874-8)
Supplement: Supplementary file 18 — Additional file 18. TRT (%) values estimated for specific brain regions (grey matter) are presented for RPM BPND and R1. [file 13550_2021_874_MOESM18_ESM.docx]

**Supplementary Table 7:** TRT (%) values estimated for specific brain regions (grey matter) are presented for RPM BP_ND_ and R_1_.

|  | **RPM BP_ND_** | | | | **RPM R_1_** | | | |
| --- | --- | --- | --- | --- | --- | --- | --- | --- |
|  | **HC** | | **AD** | | **HC** | | **AD** | |
|  | **TRT%** | **SD** | **TRT%** | **SD** | **TRT%** | **SD** | **TRT%** | **SD** |
| **Medial Temporal Lobe** | -5 | 21 | 13 | 22 | -3 | 10 | 6 | 10 |
| **Frontal Cortex** | -4 | 11 | 7 | 18 | -2 | 6 | 4 | 11 |
| **Parietal Cortex** | -7 | 12 | 7 | 15 | -2 | 6 | 3 | 11 |
| **Temporal Cortex** | -8 | 13 | 9 | 19 | -3 | 7 | 4 | 10 |
| **Occipital Cortex** | -7 | 7 | 9 | 18 | -1 | 6 | 3 | 11 |
| **Anterior Cingulate Cortex** | -6 | 17 | 5 | 11 | -4 | 6 | 0 | 5 |
| **Posterior Cingulate Cortex** | -10 | 13 | 7 | 10 | -2 | 6 | 5 | 11 |
| **Thalamus** | -5 | 10 | 15 | 25 | -2 | 8 | 3 | 11 |
| **Putamen** | -5 | 11 | 8 | 17 | -2 | 5 | 5 | 10 |
| **Caudate Nucleus** | -4 | 13 | 7 | 26 | -2 | 5 | 7 | 12 |
| **Hippocampus** | -6 | 21 | 11 | 21 | -4 | 9 | 5 | 9 |
| **Cerebellum** | -8 | 7 | 12 | 19 | -3 | 5 | 3 | 10 |
| **Brainstem** | -19 | 23 | 15 | 48 | -2 | 5 | 4 | 8 |
