# Supplementary material for: Validation and test–retest repeatability performance of parametric methods for [11C]UCB-J PET
Source: EJNMMI Res. 2022 Jan 24;12:3. doi: 10.1186/s13550-021-00874-8 (PMC8786991; doi:10.1186/s13550-021-00874-8)
Supplement: Supplementary file 19 — Additional file 19. Coefficients of determination (r2) and slopes of parametric [11C]UCB-J VT, K1 and BPND against corresponding 1T2k_VB estimates for HCs using 90 minutes data. All the Hammers ROIs were included for this analysis. [file 13550_2021_874_MOESM19_ESM.docx]

**Supplementary Table 8.** Coefficients of determination (r^2^) and slopes of parametric [^11^C]UCB-J V_T_, K_1_ and BP_ND_ against corresponding 1T2k_V_B_ estimates for HCs using 90 minutes data. All the Hammers ROIs were included for this analysis.

|  | **HC** | |
| --- | --- | --- |
|  | ***r*^2^** | **Slope** |
| **Spectral analysis^a^ V_T_** | 0.90 | 0.84 |
| **Spectral analysis ^a^ K_1_** | 0.96 | 0.87 |
| **RPM^b^ BP_ND_** | 0.90 | 0.79 |
| **SRTM2^b^ BP_ND_** | 0.88 | 0.82 |
