# Supplementary material for: Validation and test–retest repeatability performance of parametric methods for [11C]UCB-J PET
Source: EJNMMI Res. 2022 Jan 24;12:3. doi: 10.1186/s13550-021-00874-8 (PMC8786991; doi:10.1186/s13550-021-00874-8)
Supplement: Supplementary file 20 — Additional file 20. Coefficients of determination (r2) and slopes of parametric [11C]UCB-J BPND and R1 against corresponding SRTM estimates using 90 minutes data. All the Hammers ROIs were included for this analysis. [file 13550_2021_874_MOESM20_ESM.docx]

**Supplementary Table 9.** Coefficients of determination (r^2^) and slopes of parametric [^11^C]UCB-J BP_ND_ and R_1_ against corresponding SRTM estimates using 90 minutes data. All the Hammers ROIs were included for this analysis.

|  | **HC** | |
| --- | --- | --- |
|  | ***r*^2^** | **Slope** |
| **RPM^b^ BP_ND_** | 0.97 | 0.99 |
| **RPM^b^ R_1_** | 1.00 | 1.01 |
| **SRTM2^b^ BP_ND_** | 0.92 | 0.99 |
| **SRTM2^b^ R_1_** | 0.99 | 1.02 |
