# Supplementary material for: Validation and test–retest repeatability performance of parametric methods for [11C]UCB-J PET
Source: EJNMMI Res. 2022 Jan 24;12:3. doi: 10.1186/s13550-021-00874-8 (PMC8786991; doi:10.1186/s13550-021-00874-8)
Supplement: Supplementary file 21 — Additional file 21. TRT (%) values estimated for whole brain (grey matter) are presented for each parametric method using 90 minutes data. [file 13550_2021_874_MOESM21_ESM.docx]

**Supplementary Table 10.** TRT (%) values estimated for whole brain (grey matter) are presented for each parametric method using 90 minutes data.

| **TRT whole brain (grey matter)** | | |
| --- | --- | --- |
|  | **HC** | |
|  | **Mean** | **SD** |
| **SA V_T_** | -5 | 6 |
| **SA K_1_** | 0 | 12 |
| **RPM DVR (BP_ND_+1)** | -3 | 6 |
| **RPM R_1_** | -4 | 5 |
| **SRTM2 DVR (BP_ND_+1)** | 0 | 7 |
| **SRTM2 R_1_** | -3 | 9 |
